# Physical Exercise Decreases Complement‐Mediated Synaptic Loss and Protects Against Cognitive Impairment by Inhibiting Microglial Tmem9‐ATP6V0D1 in Alzheimer's Disease

**DOI:** 10.1111/acel.14496

**Published:** 2025-01-27

**Authors:** Shiyin Li, Mingyue Li, Ge Li, Lili Li, Xiaofeng Yang, Zejie Zuo, Liying Zhang, Xiquan Hu, Xiaofei He

**Affiliations:** ^1^ Department of Rehabilitation Medicine The Third Affiliated Hospital, Sun Yat‐Sen University Guangzhou Guangdong China; ^2^ Guangdong Provincial Key Laboratory of Laboratory Animals Guangdong Laboratory Animals Monitoring Institute Guangzhou Guangdong China

**Keywords:** Alzheimer's disease, complement activation, microglia, physical exercise, Tmem9

## Abstract

Physical exercise is known to slow synaptic neurodegeneration and cognitive aging in Alzheimer's disease (AD). The benefits of physical exercise are related to reduced amyloid beta (A*β*) deposition and increased synaptic plasticity. Yet little is known about the mechanisms that mediate these effects. Here, we show that physical exercise down‐regulated the microglial Tmem9 protein, inhibited C1q activation, and decreased C1q‐dependent microglial synapse engulfment, eventually ameliorating cognitive impairment in 5xFAD mice. Furthermore, using oA*β* cultured‐BV2 in vitro, we show that downregulation of microglial Tmem9 was sufficient to restrain complement activity and decrease microglia‐mediated synaptic loss, whereas overexpression of microglial Tmem9 tended to promote complement activation and induced synaptic loss, abolishing exercise‐associated protection. Finally, we show that microglial Tmem9 contributed to complement activation by regulating ATP6V0D1, a vesicular (H^+^) ATP‐dependent proton pump (V‐ATPase) subunit that regulates V‐ATPase assembly. Together, our results demonstrate that exercise is a potential treatment for AD patients. In an AD mouse model, it decreased the levels of microglial Tmem9 to inhibit the activation of complement, alleviated complement‐dependent synaptic loss, and eventually ameliorated emotional and cognitive disorders.

## Graphical abstract

In a 5xFAD mouse model, we observed that physical exercise reduced the levels of microglial Tmem9, thereby inhibiting complement activation through its interaction with ATP6V0D1. Consequently, this attenuation of complement‐dependent synaptic loss led to significant improvements in both emotional and cognitive impairments.

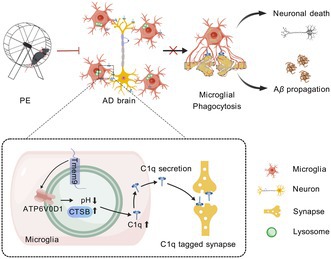

## 1 Introduction

Alzheimer's disease (AD) is the most common form of dementia, which is characterized by progressive cognitive decline. It currently affects more than 50 million people worldwide and shows growing prevalence. However, effective disease‐modifying therapies for AD are lacking (Scheltens et al. 2021). Extracellular amyloid *β* (A*β*) plaques, intracellular neurofibrillary tangles (NFTs), and related neuroinflammation are key pathological features of AD (Leng and Edison 2021). Owing to discouraging clinical trials using humanized anti‐A*β* or anti‐tau monoclonal antibodies (Song et al. 2022), it is imperative to identify a more promising treatment for AD patients. Synaptic loss, which has been correlated with cognitive impairment in AD patients, is thought to be an important target for AD treatment (Tzioras et al. 2023). Synaptic markers in cerebrospinal fluid or plasma, such as neurofilament light chain and synaptic vesicle glycoprotein 2 A, have become accepted biomarkers of AD, and are expected to improve early diagnostic efficiency and help predict the outcomes of the disease in AD patients (Stevenson‐Hoare et al. 2023; Wang, Zhang, et al. 2024). In addition, degenerated synapses promote A*β* and tau presence in remote areas, thereby exacerbating the pathologies (de Calignon et al. 2012). Understanding how synaptic loss in AD patients occurs is therefore essential for the development of new interventions.

Many studies have reported that dysfunction of neuronal mitochondria, release of neuroinflammatory cytokines and reactive oxygen species (ROS), and toxicities of soluble A*β* or tau proteins have contributed to synaptic loss in AD patients (Baik et al. 2019; Kent, Spires‐Jones, and Durrant 2020; Merlini et al. 2019). Furthermore, microglia may excessively engulf synapses, leading to synapse elimination, which is independent of A*β* or NFT pathologies. As such, microglial engulfment of synapses is indicative of early synaptic loss in AD (Taddei et al. 2023). It has been reported that overactivation of the classical complement pathway (CCP), an important innate immune molecule cascade, contributes to synapse removal during developmental circuit refinement and in AD (Dejanovic et al. 2018). C1q, the initiating factor of the CCP, binds to synapses and opsonizes the synapses, which leads to C1q‐dependent microglial synapse engulfment (Zhou et al. 2023). Blocking C1q binding is therefore sufficient to attenuate synaptic loss in AD patients (Hong et al. 2016; Zhou et al. 2023).

Despite the pivotal role of C1q in synaptic loss and neurodegeneration, it is not clear how C1q and upstream signaling are regulated in the brain. Recently, lysosomal dysfunction was reported to activate the complement pathway, leading to complement‐mediated synaptic loss (Dejanovic et al. 2022; Lui et al. 2016). As innate immune cells in the brain, microglia exert multiple effects during the progression of AD. They clear away A*β*, depending on the lysosomal degradative process, and the levels of cathepsin increased in an attempt to limit A*β*‐associated pathologies (Heckmann et al. 2019). However, phagocytosis of A*β* in microglia may damage lysosomal structures, triggering the release of lysosomal components into the cytoplasm, which is involved in the initiation of neuroinflammatory responses and synaptic losses in AD patients (Ibata et al. 2019; Tran and Silver 2021). Furthermore, lysosomal cathepsins strongly interact with the complement factor, C1q (Chen et al. 2020). Therefore, we aimed to identify strategies to inhibit the lysosome genes related to complement activation, while facilitating the degradation properties of microglia.

Physical exercise (PE) is believed to slow cognitive impairment in AD patients. It reduces neuroinflammation, rescues synaptic plasticity, and upregulates the proliferation as well as survival of hippocampal newborn cells (De Miguel et al. 2021; Lourenco et al. 2019). However, the mechanism by which exercise causes these benefits is poorly understood. In this study, we aimed to characterize the regulation of complement‐mediated synaptic loss in an AD model system and to identify the protective mechanisms of PE. To accomplish these goals, we conducted a 3‐month study of voluntary wheel running 5xFAD mice. We determined their differential gene expressions at the protein level using proteomics analyses. We found that PE inhibited complement activation and decreased complement‐mediated synaptic loss. As a result, PE promoted the survival of synaptic structures, decreased the propagation of A*β*, and eventually alleviated anxiety disorders and cognitive impairments. PE may therefore optimize the process of aging and promote a higher quality of life for AD patients.

## 2 Materials and Methods

The detailed methods for the animal treatments, proteomic analysis, intracerebroventricular (i.c.v) injection of adeno‐associated virus (AAV), immunofluorescent staining, western blotting, transmission electron microscopy (TEM), Golgi staining, enzyme‐linked immunosorbent assay (ELISA) for the C1q factor, cell culture and drug treatments, A*β*42 oligomer preparation, MTT assay, lentivirus transfection, and measurement of lysosomal pH value can be found in Supporting Information.

### 2.1 Animal Care and Approval

This study was approved by the Institutional Animal Care and Use Committee (IACUC) of Guangdong Laboratory Animal Monitoring Institute. The 5‐month‐old male 5xFAD mice and negative mice were used in this study. The mice were housed under these conditions for 3 months. All animal experimental procedures followed rules dictated by the animal ethics Committee and were designed in compliance with the ARRIVE guidelines, and no exclusion of data was done. See Supporting Information for details.

### 2.2 Behavior Experiments for Anxiety and Memory

Anxiety and exploratory activity were detected by the open field test. The object recognition test (novel object recognition) was used to test the cognition and memory functions. Morris water maze was performed to test the spatial learning and memory. See Supporting Information for details.

### 2.3 Cell Culture

The mouse BV2 microglia cell line and HT22 neuronal cell line were used in the cell experiments. See Supporting Information for details.

### 2.4 Statistical Analysis

All data were presented as means ± standard deviations (Mean ± SD), and *p* < 0.05 was considered statistically significant. See Supporting Information for details.

## 3 Results

### 3.1 PE Decreased the Levels of Tmem9 in Microglia and Ameliorated the Cognitive Impairment in AD mice

**FIGURE 1:**
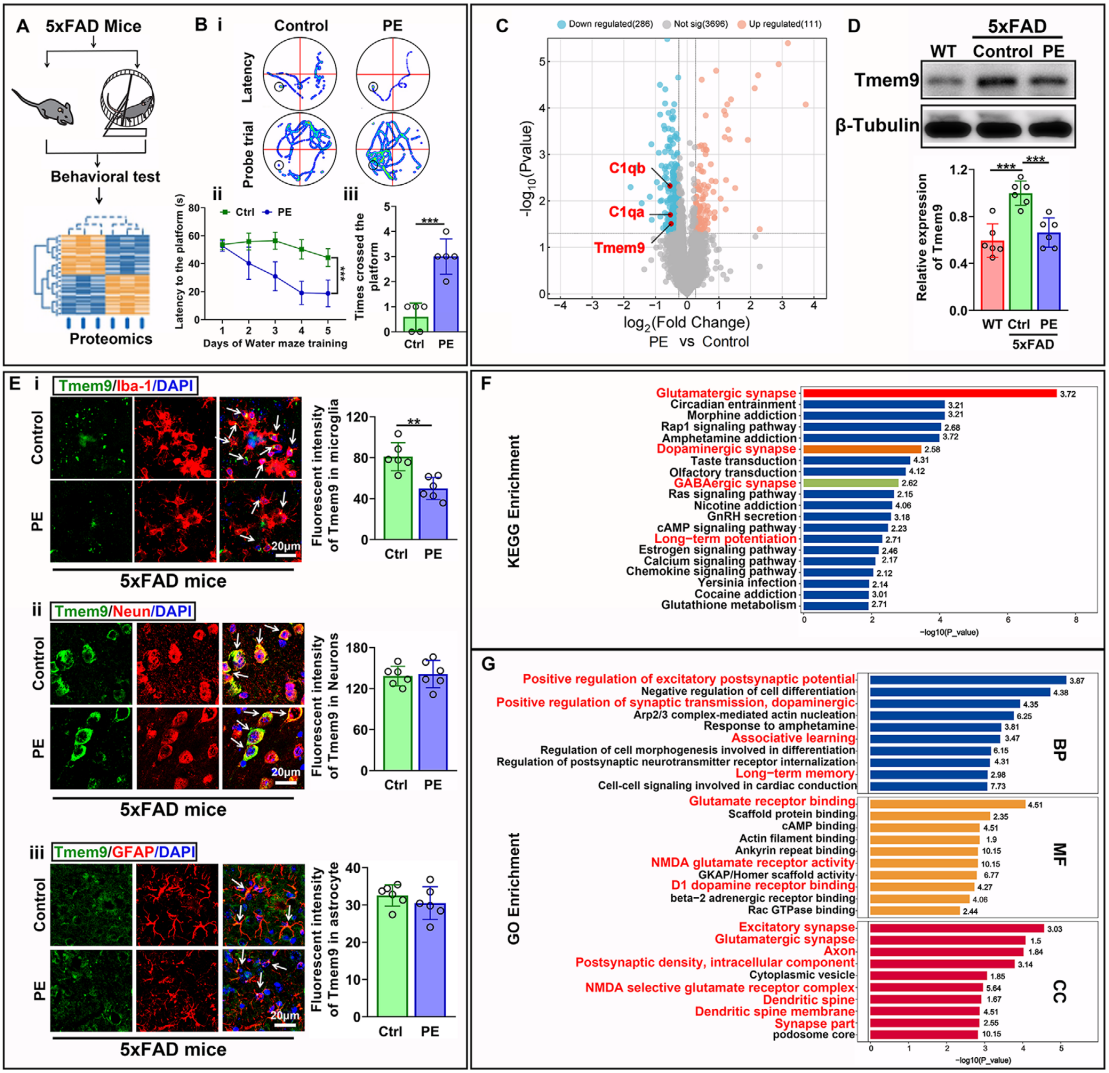
Physical exercise (PE) ameliorated cognitive impairment, decreased Tmem9 expression in microglia, and regulated synapse signaling in 5xFAD mice. (A) Experimental design for PE program implementation, proteomics analysis, and behavioral tests in the 5xFAD mouse model. (B) Travel path tracings of mice in water maze training and the probe trial during the Morris water maze (i). The latencies to the platform over a 5‐day training course (ii) and the number of target crossings (iii) in the Morris water maze. (C) Proteomics analysis of differentially expressed proteins between the PE and control groups. (D) Western blot analysis of Tmem9 expression among the WT, Control‐5xFAD (Ctrl‐5xFAD), and PE‐5xFAD groups. (E) Immunofluorescence analysis for the Tmem9 protein in microglia (i), neurons (ii), and astrocytes (iii) between the control (Ctrl) and PE‐5xFAD groups. (F) The top 20 pathways from the Kyoto Encyclopedia of Genes and Genomes enrichment analysis. (G) The top 10 pathways for the Gene Ontology enrichment analysis. The results are expressed as the mean ± SD. *p < 0.05; **p < 0.01; ***p < 0.001; ****p < 0.0001.

The Morris water maze test was used to assess the effect of PE on cognition impairment in 5xFAD mice (Figure 1A). We found that latency to the platform was significantly decreased in the PE group compared with the control group during water maze training. During the probe trial, the times crossing the former platform in the PE group increased compared with the control group, suggesting that PE improved spatial learning and memory in the model mice (Figure 1B). We then performed proteomics analyses to identify differentially expressed proteins (DEPs) between the PE and control (sedentary) groups of 5xFAD mice after 3 months of exercise (Figure 1A). Among these downregulated proteins, Tmem9 (transmembrane protein 9) accompanied by C1qa and C1qb proteins is a potential candidate for intervention targets in AD mice (Figure 1C), as synaptic loss, critically induced by C1q activation, is strongly correlated with cognitive decline in AD (Dejanovic et al. 2022, 2018; Leng and Edison 2021), and Tmem proteins regulate lysosomal acidification, thereby affecting the activation of complement cascades (Feng et al. 2021; Klein et al. 2017). Tmem9 is a type I transmembrane protein located within late endosomes/lysosomes, which has been shown to regulate vesicular acidification, lysosomal degradation and inflammatory cytokine secretion in many tissues and cells (Kveine et al. 2002; Wei et al. 2018). In our study, we used western blots to verify the downregulation of Tmem9. The level of Tmem9 protein increased in Control‐5xFAD (Ctrl‐5xFAD) mice compared with wild‐type (WT) mice and was decreased by PE in 5xFAD mice. The levels of Tmem9 showed no significant difference between the WT group and PE‐5xFAD group (Figure 1D). Cellular localization of Tmem9, using immunofluorescent staining, showed that Tmem9 proteins were all expressed in microglia, neurons, and astrocytes (Figure 1E), but the fluorescent intensity of Tmem9 in microglia was significantly decreased in the PE‐5xFAD group compared with the Ctrl‐5xFAD group. However, the fluorescent intensities of Tmem9 protein in neurons and astrocytes showed no significant difference between the PE‐5xFAD and Ctrl‐5xFAD groups (Figure 1E). Kyoto Encyclopedia of Genes and Genomes (KEGG) enrichment analysis showed that PE activated the signaling pathway related to glutamatergic, dopaminergic, and GABAergic synapses (Figure 1F). Gene Ontology (GO) enrichment analysis showed that DEPs were enriched in signaling related to positive regulation of excitatory postsynaptic potential and synaptic transmission, long‐term memory, glutamate receptor binding and activity, excitatory synapse, axons, and dendritic spine membrane in the AD brain (Figure 1G). These results suggested that PE decreased the levels of microglial Tmem9 protein, regulated synaptic function, and attenuated the cognitive impairment in the AD mouse model.

### 3.2 PE Protected Against Anxiety and Cognitive Impairment in an AD Mouse Model by Decreasing Levels of Microglial Tmem9 Protein

**FIGURE 2:**
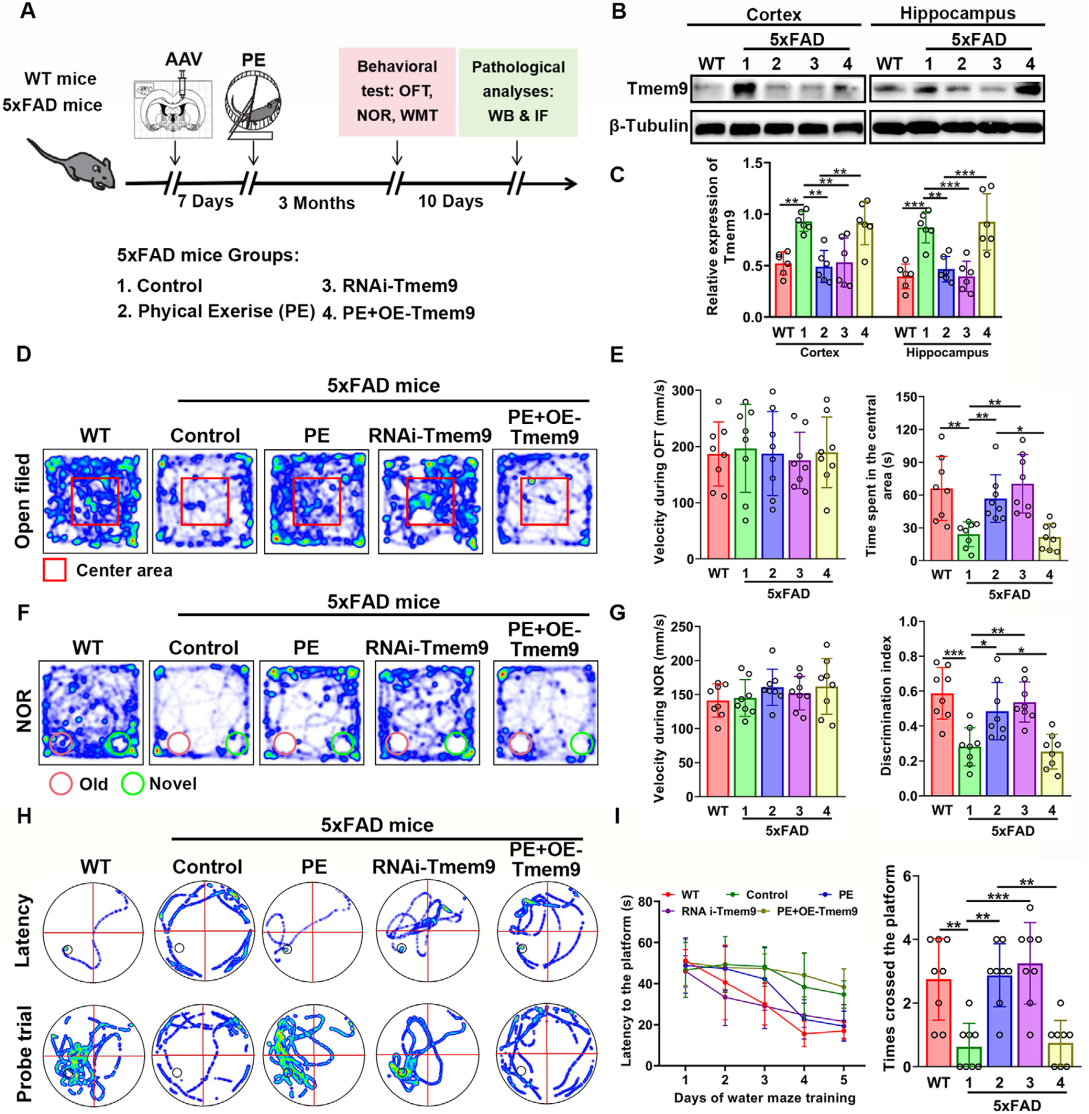
Physical exercise (PE) ameliorated emotional and cognitive dysfunctions via decreasing levels of microglial Tmem9. (A) Experimental design for adeno‐associated virus (RNAi‐Tmem9/OE‐Tmem9‐AAVs) injection, PE program implementation, behavioral tests, and pathological analyses of the 5xFAD mice model. (B, C) Western blot analysis of Tmem9 expression among the WT, Ctrl‐5xFAD, PE‐5xFAD, RNAi‐Tmem9, and PE + OE‐Tmem9 groups. (D) Travel path tracings of mice during the open field test. (E) Velocity and time spent in the central area during the open field test. (F) Travel path tracings of mice during novel object recognition. (G) Velocity and discrimination index of novel object recognition. (H) Travel path tracings of mice during water maze training and probe trials in the Morris water maze. (I) Latencies to the platform over a 5‐day training course and times of target crossings in the Morris water maze. The results are expressed as the mean ± SD. *p < 0.05; **p < 0.01; ***p < 0.001; ****p < 0.0001.

To determine the possible involvement of microglial Tmem9 in the regulation of synaptic dysfunction in AD model mice, as well as PE‐associated protection of synapses, we injected AAV containing a F4/80 promoter (Wang, Wang, et al. 2024) to overexpress microglial Tmem9 in PE‐5xFAD mice. We also injected interference RNA (RNAi‐Tmem9‐AAV) into Ctrl‐5xFAD mice to investigate whether PE‐associated protection could be mimicked by microglial Tmem9 knockdown (Figure 2A). Western blots showed that the level of Tmem9 proteins was significantly increased in Ctrl‐5xFAD mice compared with WT mice, which was significantly decreased by PE and by injection of RNAi‐Tmem9‐AAV. Furthermore, the level was increased in the PE + overexpressing (OE)‐Tmem9‐AAV group compared with PE‐5xFAD mice (Figure 2B,C). Immunofluorescent staining showed there was no GFP expression in the WT, Ctrl‐5xFAD, or PE‐5xFAD groups (Figure S1A–C). Furthermore, AAV‐GFP expressions in the RNAi‐Tmem9 and PE + OE‐Tmem9 groups were co‐localized within Iba‐1 microglia (Figure S1D,E), but not in neurons or astrocytes (Figure S2).

Anxiety behavior was then examined using the open field test (Figure 2D,E), which showed that the velocities were not significantly different among these five groups, while the time spent in the central area decreased in Ctrl‐5xFAD mice compared with the WT group, indicating anxiety‐like behavior in the AD model mice. Compared to Ctrl‐5xFAD mice, the time significantly increased in the PE and RNAi‐Tmem9 groups and showed no difference between the PE and RNAi‐Tmem9 groups. However, the time decreased in the PE + OE‐Tmem9 group compared with the PE group, but did not differ from that for the Ctrl‐5xFAD group. Together, these results suggest that PE attenuated anxiety behavior in the AD model mice. This result was mimicked by RNAi‐Tmem9‐AAV injection, whereas it was abolished by overexpression of microglial Tmem9.

Mice have an innate preference for novelty. They tend to spend more time exploring a new object to provide information to remember it, which is termed recognition ability. We therefore conducted a new object recognition test to determine the effect of PE on cognition in AD model mice. As shown in Figure 2F,G, the velocities were not significantly different among these five groups, while the discrimination index decreased in Ctrl‐5xFAD mice compared with WT mice, indicating cognitive impairment. Compared to Ctrl‐5xFAD mice, discrimination indices were significantly increased in the PE‐5xFAD and RNAi‐Tmem9 groups, but they showed no difference between the PE and RNAi‐Tmem9 groups. However, the discrimination index was decreased in the PE + OE‐Tmem9 group, when compared with the PE group, but did not differ from the Ctrl‐5xFAD group (Figure 2F,G). During water maze training, latencies at the platform showed no significant differences among these five groups (Figure 2H,I), but during the probe trial, the times crossing the former platform was decreased in Ctrl‐5xFAD mice compared WT mice, indicating spatial memory impairment in AD model mice. Compared to Ctrl‐5xFAD mice, times were significantly increased in the PE‐5xFAD and RNAi‐Tmem9 groups, but showed no difference between the PE and RNAi‐Tmem9 groups. However, the times decreased in the PE + OE‐Tmem9 group compared with the PE group (Figure 2H,I). Together, these results indicate that PE attenuated cognitive impairment in AD model mice, which could be abolished by overexpression of microglial Tmem9, and that knockdown of microglial Tmem9 mimicked the PE‐associated protection.

### 3.3 PE Decreased C1q‐Mediated Microglial Engulfment of Synapses by Decreasing Tmem9 Expression

**FIGURE 3:**
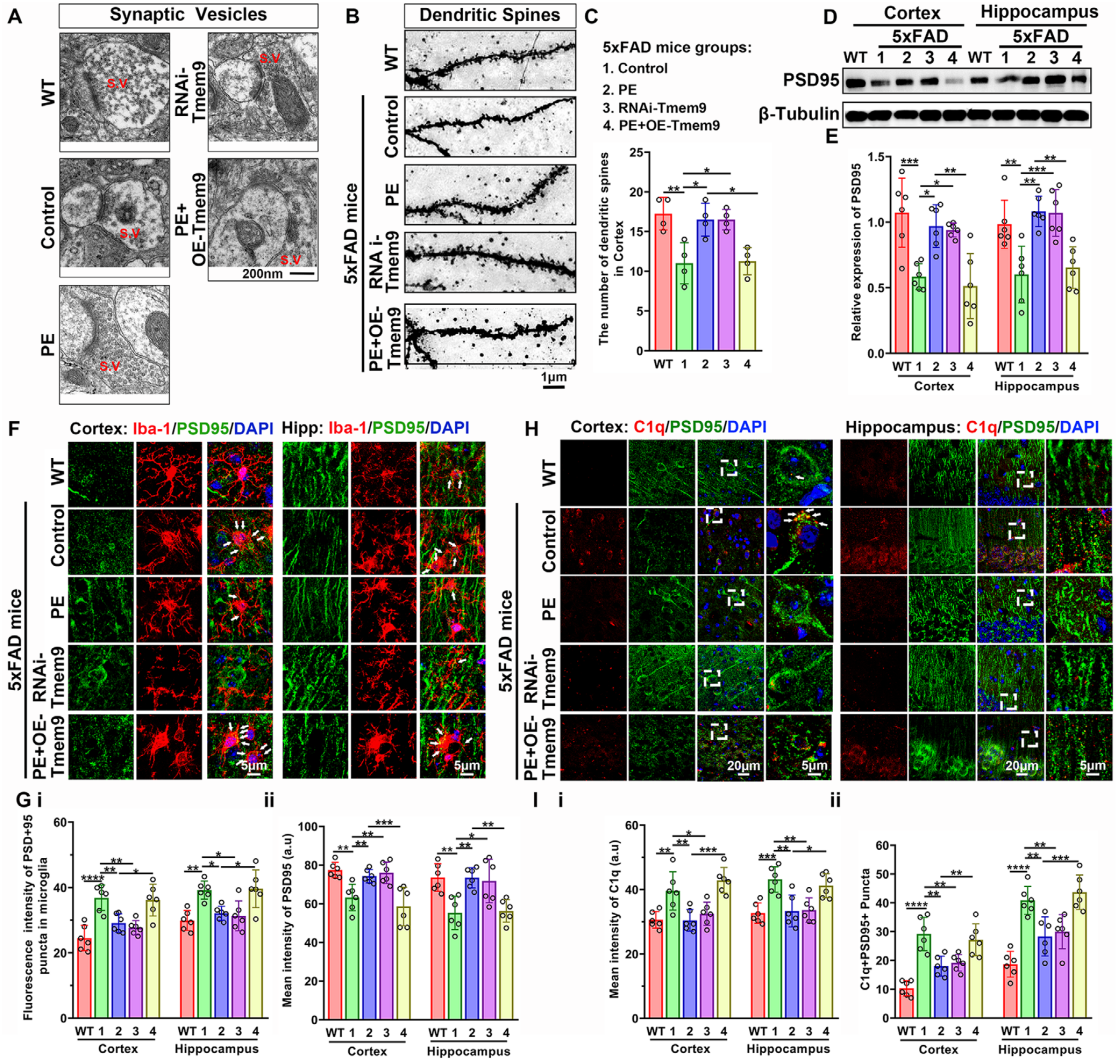
Physical exercise (PE) increased synaptic vesicles and the density of dendritic spines, via decreasing microglial engulfment of C1q‐tagged synapse 5xFAD mice. (A) Transmission electron microscopy of synaptic vesicles (red arrows) for the WT, Ctrl‐5xFAD, PE‐5xFAD, RNAi‐Tmem9, and PE + OE‐Tmem9 groups. Bar = 200 nm. (B, C) Golgi staining images and analyses of dendritic spines in neurons among the WT, Ctrl‐5xFAD, PE‐5xFAD, RNAi‐Tmem9, and PE + OE‐Tmem9 groups. Scale bar = 1 μm. (D, E) Western blot analysis for PSD95 expressions among the WT, Ctrl‐5xFAD, PE‐5xFAD, RNAi‐Tmem9, and PE + OE‐Tmem9 groups. (F, G) Immunofluorescent analysis of the PSD95 in microglia among the WT, Ctrl‐5xFAD, PE‐5xFAD, RNAi‐Tmem9, and PE + OE‐Tmem9 groups. (H, I) Immunofluorescent analysis for C1q + PSD95+ puncta among the WT, Ctrl‐5xFAD, PE‐5xFAD, RNAi‐Tmem9, and PE + OE‐Tmem9 groups. The results are expressed as the mean ± SD. *p < 0.05; **p < 0.01; ***p < 0.001; ****p < 0.0001.

As mentioned above, KEGG analysis showed that the DEPs between the control and PE groups of 5xFAD mice were enriched in the signaling pathways related to synaptic function (Figure 1F). We therefore used TEM and Golgi staining to examine the effect of PE on synaptic structures in 5xFAD mice, as well as the regulation of microglial Tmem9. The TEM results showed that there were numerous synaptic vesicles in WT mice, which were less in Ctrl‐5xFAD mice, and the synaptic vesicles in the PE‐5xFAD and RNAi‐Tmem9 groups were more than those in the Ctrl‐5xFAD group. However, the number of synaptic vesicles in the PE + OE‐Tmem9 group was much less than that in the PE group (Figure 3A). Golgi staining and western blots showed that the number of dendritic spines in the cortex and the expression levels of postsynaptic density protein 95 (PSD95) in the cortex and hippocampus were significantly decreased in the Ctrl‐5xFAD group compared with the WT group. Compared to Ctrl‐5xFAD mice, the number and the levels significantly increased in the PE‐5xFAD and RNAi‐Tmem9 groups, which showed no difference between the PE and RNAi‐Tmem9 groups. However, the number and the levels were significantly decreased in the PE + OE‐Tmem9 group, when compared with the PE group, which did not differ from the Ctrl‐5xFAD group (Figure 3B–E). These results indicated that synaptic loss occurred in 5xFAD mice and was ameliorated by PE and injection of RNAi‐Tmem9‐AAV. Furthermore, the PE‐associated protection from synaptic loss was abolished by overexpression of microglial Tmem9.

It is also noteworthy that our differential expression analysis showed that C1qa and C1qb were also downregulated when Tmem9 was downregulated (Figure 1C). C1qa and C1qb are polypeptide chains of C1q, which have been reported to pathologically “tag” synapses, to induce aberrant phagocytosis of synapses by binding to the complement receptors on the surface of other microglia (Dejanovic et al. 2022; Leng and Edison 2021). We therefore examined microglial engulfment of synapses, as well as activation of C1q. Figure 3F,G shows that the intensities of PSD95‐labeled puncta in microglia (PSD95 + Iba1+) significantly increased and total intensities of PSD95 decreased in the Ctrl‐5xFAD group compared with the WT group. PE and RNAi‐Tmem9 treatments significantly decreased puncta intensity and increased total intensity in 5xFAD mice compared with the Ctrl‐5xFAD group. However, compared to the PE group, puncta intensity was significantly increased and total intensity was decreased in the PE + OE‐Tmem9 group, but did not differ from the control group. Figure 3H,I shows that the mean intensity of C1q and C1q+PSD95+ puncta significantly increased in the Ctrl‐5xFAD mice compared to the WT group. Compared to the Ctrl‐5xFAD mice, the intensity and puncta significantly decreased in the PE‐5xFAD and RNAi‐Tmem9 groups. However, the intensity and puncta were increased in the PE + OE‐Tmem9 group compared with the PE group, which did not differ from the Ctrl‐5xFAD mice.

To investigate the regulation of microglial Tmem9 in C1q‐mediated synapse engulfment, we treated the BV2 cells with oA*β* or LPS (Lipopolysaccharides) + oA*β* to establish the AD model in vitro and co‐cultured these BV2 cells with HT22 neuronal cells (Figure S3A). The MTT assay results showed that inhibition of BV2 cell growth was increased with increasing oA*β* concentrations, so we treated BV2 cells with 5μM oA*β* in subsequent experiments, because the inhibition on cell growth was less than 50% (Figure S4). We overexpressed or knocked down levels of Tmem9 using lentiviral transfection to examine the effects of Tmem9 on microglial engulfment of synapses. Overexpression or knockdown of Tmem9 was confirmed by RT‐qPCR (Figure S5A,B). We selected ShRNA‐f‐Tmem9‐LV for subsequent experiments because of its higher interfering efficiency, and the fluorescent results verified that GFP was expressed in all three groups (Figure S5C).

To examine C1q‐tagged PSD95 puncta in microglia, we conducted immunofluorescent staining of PSD95 and C1q, using GFP‐tagged antibodies as indicators for BV2 microglia (gray color). As shown in Figure S3B–E, in cells with negative control‐lentivirus (LV) transfection and OE‐Tmem9‐LV transfection, oA*β* incubation increased the number of C1q + PSD95+ puncta in GFP+ positive microglia and was further increased in the LPS + oA*β* group. However, in cells with ShRNA‐Tmem9 transfection, the number showed no significant differences in the sham, oA*β*, and LPS + oA*β* groups (Figure S3D,E). Among the cells with oA*β* or LPS + oA*β* incubation, C1q + PSD95+ puncta in GFP+ microglia were significantly increased in the OE‐Tmem9 group compared with cells in the negative control group, whereas puncta were significantly decreased in the ShRNA‐Tmem9 group (Figure S3E). For negative control and OE‐Tmem9 lentivirus transfection cells, the total fluorescent intensity of PSD95 was significantly decreased in the oA*β* group, and the intensity was further decreased in the LPS + oA*β* group. However, for cells with ShRNA‐Tmem9 lentivirus transfection, the intensity was decreased in the LPS + oA*β* group compared to the sham groups (Figure S3F). Together, these results indicated that microglia engulfed the C1q‐tagged synapses, depending on the level of Tmem9, leading to synaptic loss in AD, both in vivo and in vitro. PE decreased the numbers of C1q‐tagged synapses and inhibited excessive engulfment of microglia, thereby increasing synaptic survival. Furthermore, knockdown of microglial Tmem9 mimicked PE‐associated protection, whereas overexpression of microglial Tmem9 blocked PE‐associated protection.

### 3.4 PE Inhibited Activation of C1q Complement by Decreasing Levels of Microglial Tmem9, Both In Vivo and In Vitro

**FIGURE 4:**
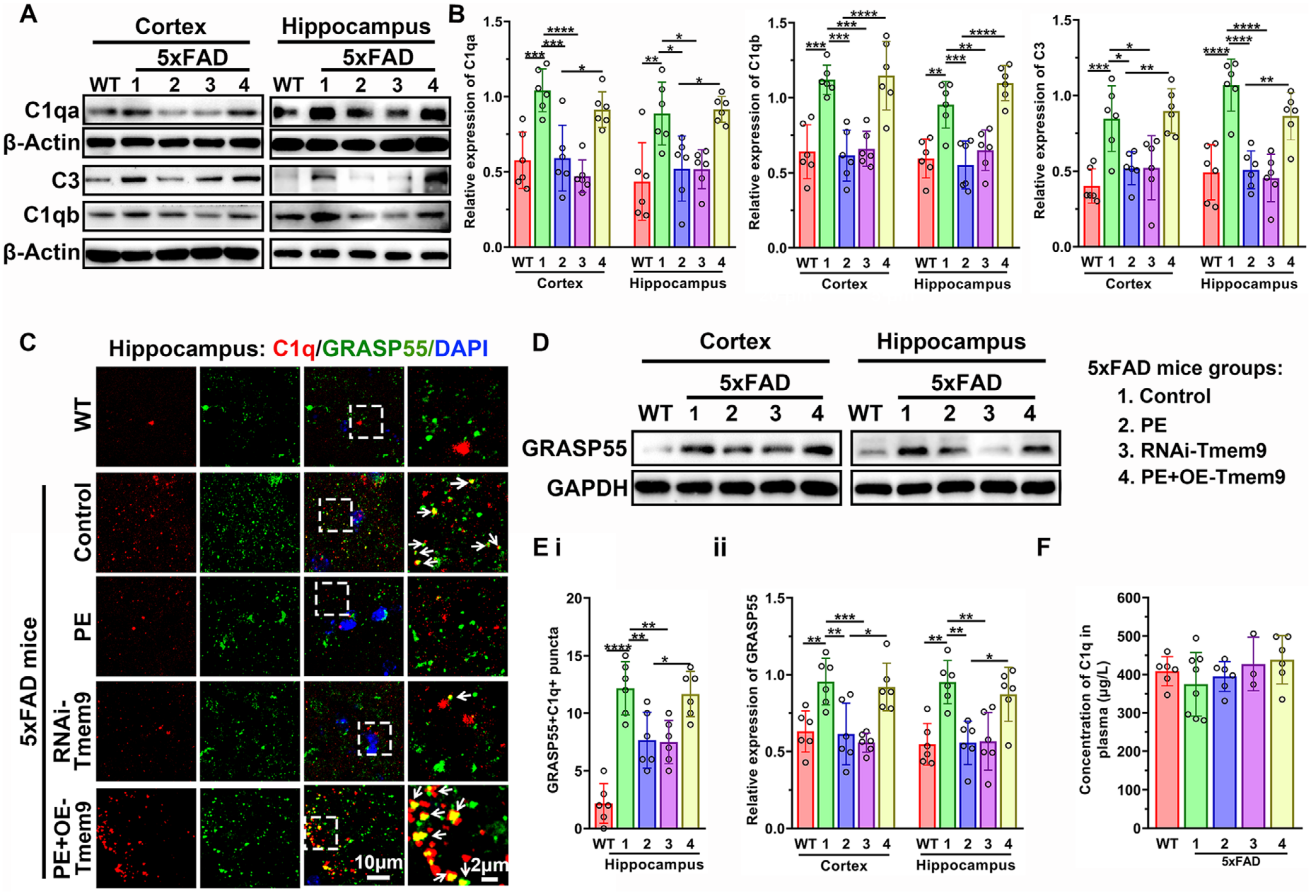
Physical exercise (PE) decreased the activation and secretion of C1q complement by inhibiting microglial Tmem9. (A, B) Western blot analysis for the expressions of C1qa, C1qb, and C3 among the WT, Ctrl‐5xFAD, PE‐5xFAD, RNAi‐Tmem9, and PE + OE‐Tmem9 groups. (C) Representative confocal images of C1q + GRASP55+ puncta in the hippocampus among the WT, Ctrl‐5xFAD, PE‐5xFAD, RNAi‐Tmem9, and PE + OE‐Tmem9 groups. (D) Chemiluminescent images of GRASP55 in the cortex and hippocampus among the WT, Ctrl‐5xFAD, PE‐5xFAD, RNAi‐Tmem9, and PE + OE‐Tmem9 groups. (E) Comparison analysis for C1q + GRASP55+ puncta (i) and western blot analysis for the GRASP55 protein expressions among the WT, Ctrl‐5xFAD, PE‐5xFAD, RNAi‐Tmem9, and PE + OE‐Tmem9 groups (ii). (F) Enzyme‐linked immunosorbent assay of plasma C1q levels among the WT, Ctrl‐5xFAD, PE‐5xFAD, RNAi‐Tmem9, and PE + OE‐Tmem9 groups. The results are expressed as the mean ± SD. *p < 0.05; **p < 0.01; ***p < 0.001; ****p < 0.0001.

Activated microglia produce and secrete complement, C1q, leading to synapse elimination (Liddelow et al. 2017; Lui et al. 2016). We examined the regulation of microglial Tmem9 on C1q activation in 5xFAD mice, as well as the regulation mechanism of PE. Because C1q is the initiating factor of the CCP, we also examined its downstream complement factor, C3. As expected, western blot results showed that C1qa, C1qb, and C3 levels were significantly increased in Ctrl‐5xFAD mice, when compared with the WT group. Compared to the Ctrl‐5xFAD group, the expressions significantly decreased in the PE‐5xFAD and RNAi‐Tmem9 groups. However, the expressions were significantly increased in the PE + OE‐Tmem9 group compared with the PE group (Figure 4A,B).

We then performed immunofluorescent co‐staining of C1q and a secretory marker, Grasp55, complement proteins that could be secreted into the extracellular space from lysosomes to label the synapses (Lui et al. 2016; Zhou et al. 2023). As shown in Figure 4C–E, the number of Grasp55 + C1q + puncta in the hippocampus and the expression levels of Grasp55 both in cortex and hippocampus were increased in the Ctrl‐5xFAD group compared with the WT group. Compared to the Ctrl‐5xFAD group, PE and RNAi‐Tmem9‐AAV treatments reduced the puncta and the levels. However, the number and the levels were significantly increased in the PE + OE‐Tmem9 group compared with the PE group, which did not differ from the control group. The ELISA results showed that C1q levels in the plasma were not significantly different among the five groups (Figure 4F).

In BV2 cells without lentivirus transfection (blank groups) and the groups with negative control or OE‐Tmem9‐lentivirus transfection, immunofluorescent staining results showed that oA*β* incubation increased the C1q intensity in microglia, which was further increased in cells with LPS + oA*β* treatment. However, in BV2 cells with ShRNA‐Tmem9 transfection, the C1q intensities showed no significant differences among the sham, oA*β*, and LPS + oA*β* groups (Figure S6A,B). These results suggested that C1q in the brain was activated and was secreted from microglia in the AD mouse brain, and that PE inhibited the activation of C1q factors, which could be mimicked by knockdown of microglial Tmem9. However, overexpression of microglial Tmem9 blocked PE‐associated regulation and activation of C1q factors.

### 3.5 PE Decreased Lysosomal Accumulation in Microglia and Inhibited Activation of Cathepsin B (CtsB) by Decreasing Microglial Tmem9

**FIGURE 5:**
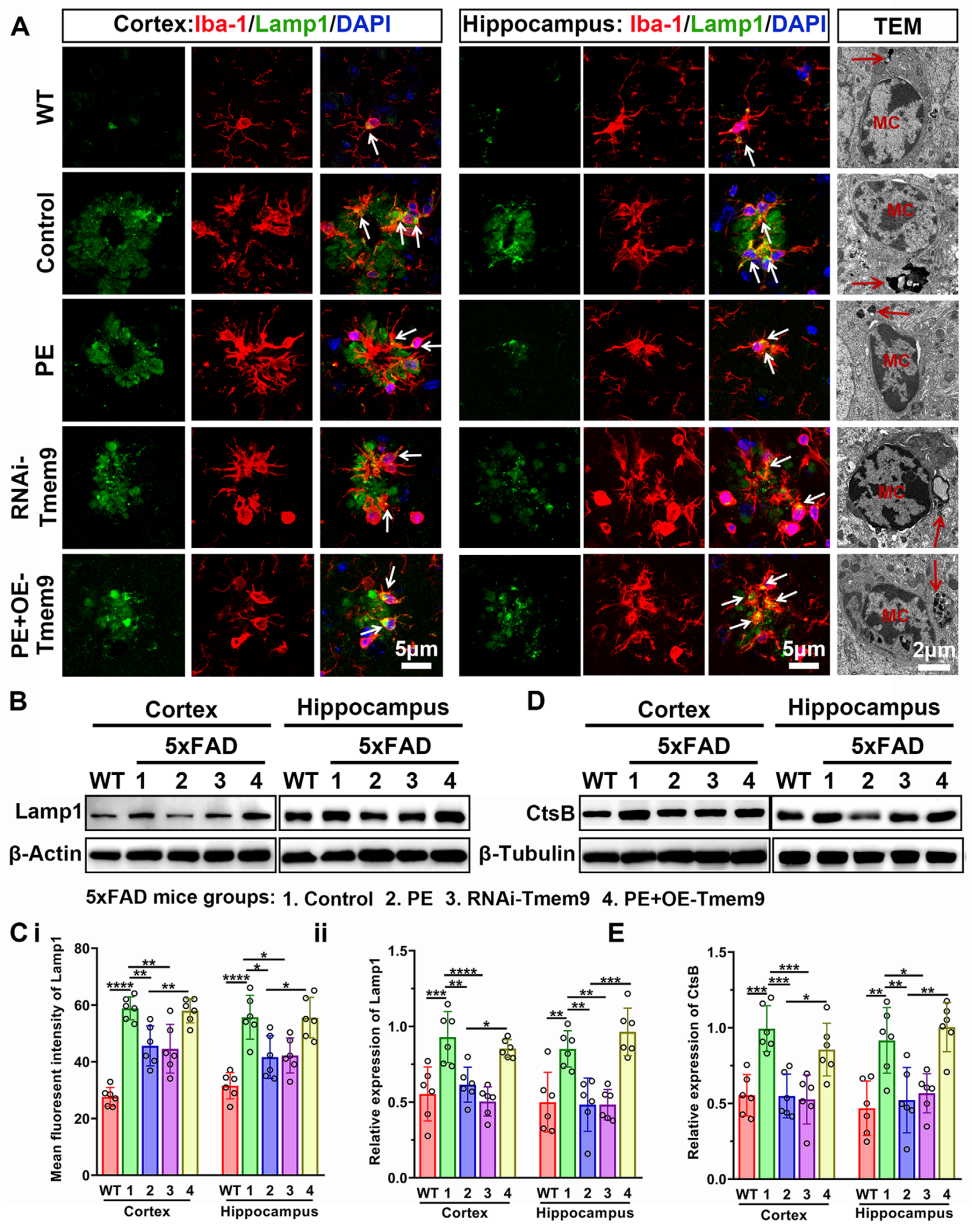
Physical exercise (PE) decreased lysosomal accumulation in microglia and inhibited activation of cathepsin B by decreasing microglial Tmem9. (A) Representative confocal images of Lamp1‐positive lysosomes in the WT, Ctrl‐5xFAD, PE‐5xFAD, RNAi‐Tmem9, and PE + OE‐Tmem9 groups, with transmission electron microscopy images showing lysosomes in microglia (white arrows). (B) Chemiluminescent images of the Lamp1 expressions in the WT, Ctrl‐5xFAD, PE‐5xFAD, RNAi‐Tmem9, and PE + OE‐Tmem9 groups. (C) Comparison analysis for Lamp1 intensities (i) and expressions (ii) among the WT, Ctrl‐5xFAD, PE‐5xFAD, RNAi‐Tmem9, and PE + OE‐Tmem9 groups. (D, E) Western blot analysis of cathepsin B expression in the cortex and hippocampus among the WT, Ctrl‐5xFAD, PE‐5xFAD, RNAi‐Tmem9, and PE + OE‐Tmem9 groups. The results are expressed as the mean ± SD. *p < 0.05; **p < 0.01; ***p < 0.001; ****p < 0.0001.

Lysosomal dysfunction is reported to be responsible for cleavage and secretion of complement factors, which are necessary for complement cascade activation (Lui et al. 2016). We therefore characterized PE‐associated effects on lysosomal function in microglia using Lamp1 (lysosome‐associated membrane protein 1). As shown in Figure 5A–C, there were increased fluorescent intensity and expression level of Lamp1 in Ctrl‐5xFAD mice compared with WT mice. PE or RNAi‐Tmem9‐AAV treatment significantly decreased the intensity and the level compared with Ctrl‐5xFAD mice, which showed no differences between the PE and RNAi‐Tmem9 groups. However, OE‐Tmem9‐AAV injection blocked the PE‐associated effects on Lamp1 intensity and level and showed no difference between the control and PE + OE‐Tmem9 groups. Moreover, regarding the control and PE + OE‐Tmem9 groups, there was lysosomal accumulation in microglia as detected by TEM, and the lysosomal accumulation was much less in the PE and RNAi‐Tmem9 treatment groups. C1q proteins were reported to be released, and this was accompanied by cathepsin B (CtsB) release (Ibata et al. 2019). Western blot results showed that the level of CtsB was increased in the 5xFAD group compared with the WT group. CtsB was decreased by PE or Tmem9‐RNAi‐AAV treatment. Overexpression of microglial Tmem9 abolished the effect of PE compared with the PE group. These results suggested that PE prevented microglial lysosomal accumulation and inhibited hyperactivation of lysosomal CtsB in the brains of AD model mice (Figure 5D,E).

### 3.6 PE Inhibited Lysosomal Accumulation and Lysosomal Over‐Acidification via Microglial Tmem9‐ATP6V0D1 Signaling, Thereby Suppressing the Amyloid Beta Deposition

**FIGURE 6:**
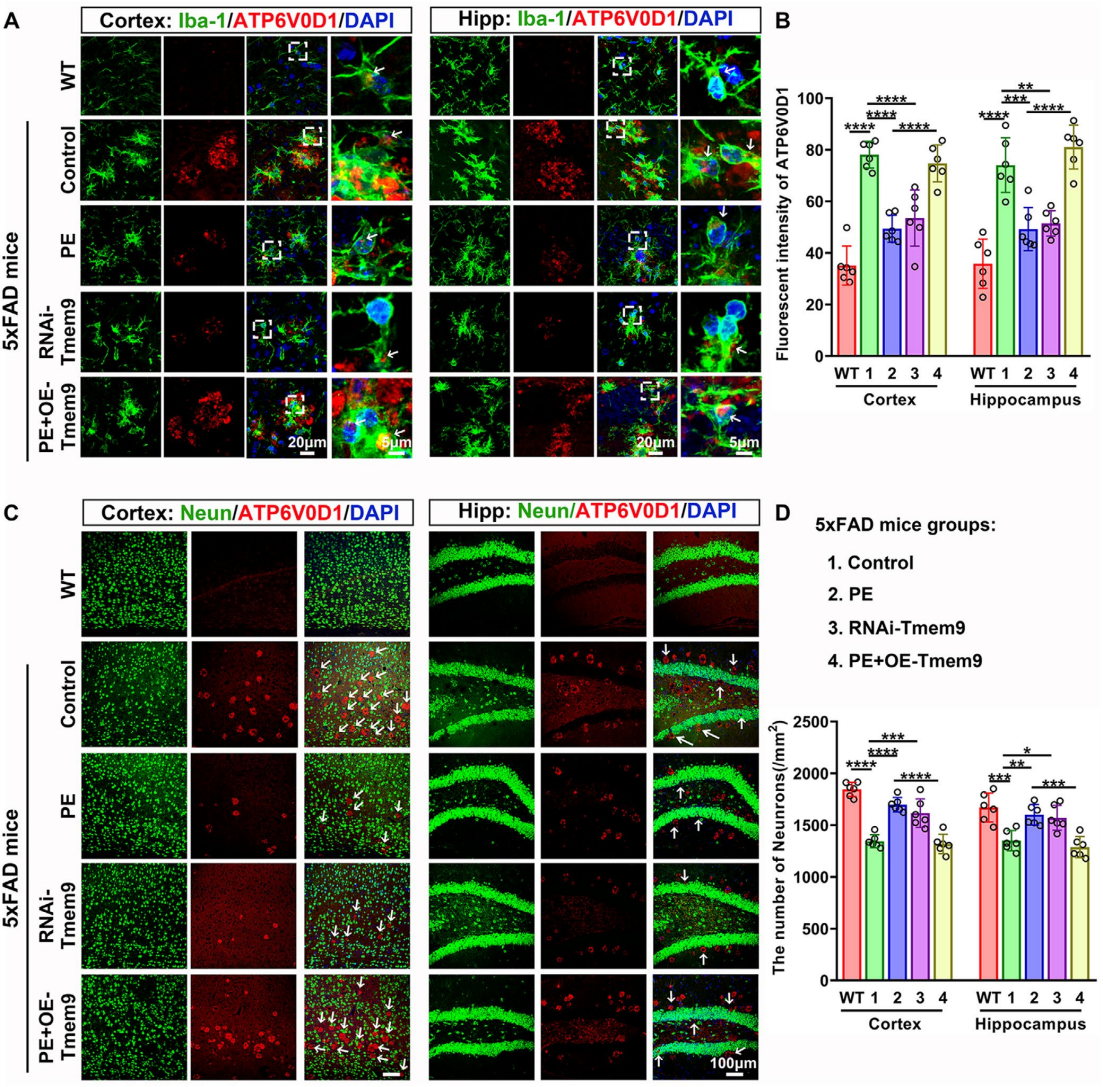
Physical exercise (PE) inhibited lysosomal accumulation in microglia and decreased neuronal loss by Tmem9‐ATP6V0D1 signaling. (A) Representative confocal images for ATP6V0D1‐positive vesicles surrounding microglia, for both the intracellular and extracellular spaces, among the WT, Ctrl‐5xFAD, PE‐5xFAD, RNAi‐Tmem9, and PE + OE‐Tmem9 groups. Right panel: Magnified images from the white dashed boxed area in left panel; the white arrow indicates the ATP6V0D1 positive vesicles in the microglia. (B) Immunofluorescent analysis for ATP6V0D1 among the WT, Ctrl‐5xFAD, PE‐5xFAD, RNAi‐Tmem9, and PE + OE‐Tmem9 groups. (C) Representative immunofluorescent images for the ATP6V0D1 and neurons, showing ATP6V0D1‐induced neuronal loss (white arrows), among the WT, Ctrl‐5xFAD, PE‐5xFAD, RNAi‐Tmem9, and PE + OE‐Tmem9 groups. (D) Comparison analysis of the number of neurons among the WT, Ctrl‐5xFAD, PE‐5xFAD, RNAi‐Tmem9, and PE + OE‐Tmem9 groups. The results are expressed as the mean ± SD. *p < 0.05; **p < 0.01; ***p < 0.001; ****p < 0.0001.

Tmem9 promotes vesicular acidification and regulates lysosomal cathepsin activities by facilitating v‐ATPase assembly (Jung et al. 2018, 2021). We detected the regulatory effects of Tmem9 on microglial V‐ATPase using antibody against ATP6V0D1 (a major subunit of V‐ATPase assembly). As shown in Figure 6A,B, the ATP6V0D1 intensity was at low levels in the WT mice brains. However, ATP6V0D1 intensities were increased in the Ctrl‐5xFAD mice compared with the WT group. ATP6V0D1 intensities were significantly decreased in the PE and RNAi‐Tmem9 groups compared with the Ctrl‐5xFAD group. However, the ATP6V0D1 intensities were increased in the PE + OE‐Tmem9 group compared with the PE group, showing no differences between the control and PE + OE‐Tmem9 groups. Furthermore, in the control and PE + OE‐Tmem9 groups, a majority of ATP6V0D1 positive vesicles leaked out and proteins were localized outside microglial cells (Figure 6A, white arrows).

Neurons were decreased in areas accumulating ATP6V0D1‐positive vesicles (Figure 6C). Statistically, the number of neurons in the Ctrl‐5xFAD group significantly decreased compared with those in the WT group. However, the number increased in the PE and RNAi‐Tmem9 treatment group compared with the control group. The number in the PE + OE‐Tmem9 group was decreased compared with that in the PE group, which did not differ from that in the control group (Figure 6C,D).

In vitro, compared to the sham group, oA*β* incubation increased the expression of ATP6V0D1 in cells with blank, negative‐ctrl‐LV, or OE‐Tmem9‐LV treatments, which was further enhanced by LPS stimulation. However, the expressions of ATP6V0D1 did not differ among the sham, oA*β*, and LPS + oA*β* groups in cells with shRNA‐Tmem9‐LV transfection. For cells with oA*β* or LPS + oA*β* incubation, the ATP6V0D1 intensity in the negative‐ctrl‐LV group did not differ from that in the blank group, but was increased in the OE‐Tmem9‐LV group and was decreased by knockdown of Tmem9 (Figure S7A,B).

We also detected the lysosomal pH values in BV2 microglia using LysoView, an acid indicator indicating acidification homeostasis in microglial lysosomes, according to the manufacturer's instructions. A higher intensity of LysoView represents a lower pH value and more acidic vesicles. As shown in Figure S7C,D, for cells without LV transfection (blank) and cells with negative‐ctrl‐LV, or OE‐Tmem9‐LV transfection, the intensities of LysoView in the oA*β* group were increased compared with the sham group, which were further enhanced in the LPS + oA*β* group. However, in cells with ShRNA‐Tmem9‐LV transfection, oA*β* or LPS + oA*β* incubation did not affect the intensities of LysoView. For cells with oA*β* or LPS + oA*β* incubation, the intensity of LysoView in the negative‐ctrl‐LV group did not differ from that in the blank group, but was increased in the OE‐Tmem9‐LV group and was decreased by knockdown of Tmem9.

We also detected amyloid beta deposition in vivo and in vitro. As shown in Figure S8A,B, compared to the Ctrl‐5xFAD group, PE and RNAi‐Tmem9‐AAV treatment reduced the total area of A*β* plaque and the percentage of A*β* whose diameter more than 20 μM. However, the total areas and the percentage were significantly increased in the PE + OE‐Tmem9 group compared with the PE group. In BV2 cells, compared to the oA*β* group, LPS and oA*β* incubation increased A*β*1‐42 intensities in cells with blank, negative‐ctrl‐LV, or OE‐Tmem9‐LV transfections, but not in cells with ShRNA‐Tmem9‐LV transfection (Figure S8C,D). For cells with oA*β* or LPS + oA*β* incubation, the A*β*1‐42 intensities in the blank group did not differ from those in the negative‐ctrl‐LV group, but they were increased in the OE‐Tmem9‐LV group and were decreased by knockdown of Tmem9 (Figure S8C,D).

## 4 Discussion

We found that PE inhibited activation and secretion of C1q complement, decreased C1q‐tagged synapses, and inhibited microglial engulfment of synapses. As a result, PE decreased synaptic loss, inhibited deposition of A*β*, and eventually mitigated anxiety disorders and cognitive impairments in AD model mice. Mechanistically, we identified microglial Tmem9 as a key regulator for the activation of C1q and microglial engulfment, as well as abnormal synaptic loss in AD model mice. PE exerts these protective effects by downregulating microglial Tmem9.

The most important finding in our study was that PE mitigated anxiety disorders and cognitive impairments by inhibiting C1q‐mediated synaptic loss. As the initiating factor of the CCP, C1q binds to synapses upon activation of the downstream cascade, leading to engulfment of the complement‐bound synapses, by microglia (Dejanovic et al. 2018, 2022). We found C1q levels in plasma were not increased in 5xFAD mice, which were not affected by PE, showing that C1q complement‐induced synaptic loss in AD involved microglia. A previous study reported that the levels of C1q were increased in cerebrospinal fluid‐derived extracellular vesicles in AD (Chatterjee et al. 2023), and that microglia induced neurotoxic reactive astrocytes, leading to cell death in neurodegenerative disorders via secreting C1q (Liddelow et al. 2017).

The second most important finding in our study was that PE inhibited the activation and secretion of C1q complement factor by decreasing levels of microglial Tmem9, which may be due to the alleviation of lysosomal dyshomeostasis. Complement activation has been reported to be strongly correlated with lysosomal cathepsins, including CtsB, CtsS, and CtsD. Lysosomal dyshomeostasis has been attributed to the cleavage and secretion of complement factors, which induce synaptic loss (Chen et al. 2020). Microglial Tmem9, decreased by PE or RNAi‐Tmem9‐AAV, may mediate the secretion of C1q by inhibiting CtsB, because the C1q family proteins are co‐released with CtsB via lysosomal exocytosis (Ibata et al. 2019). Consistently, we confirmed that the increase of CtsB levels in AD model mice brains was inhibited by exercise or Tmem9 depletion, whereas PE‐associated effects were abolished by Tmem9 overexpression. Furthermore, C1q levels in the plasma in 5xFAD mice did not differ from those in WT mice, nor were C1q levels affected by PE or Tmem9 depletion, suggesting that C1q originated from the brain.

Our results suggested that PE alleviated microglial lysosomal dyshomeostasis, perhaps by interacting with the vacuolar‐type ATPase (v‐ATPase) to inhibit lysosomal hyperacidification in microglia. We further confirmed that downregulation of Tmem9 in PE‐treated mice or shRNA‐treated cells both reduced the levels of the ATPase H+ transporting V0 subunit D1 (ATP6V0D1), a member of the V‐ATPases, and this was accompanied by higher lysosomal pH values in BV2 microglia. However, overexpression of microglial Tmem9 upregulated ATP6V0D1 levels and promoted lysosomal acidification. As a type I transmembrane protein expressed in late endosomes and lysosomes (Kveine et al. 2002), Tmem9 has been reported to accelerate the assembly of the V0 and V1 subunits of v‐ATPase, by directly binding to them, resulting in vesicular acidification (Jung et al. 2018; Bouché et al. 2016; Mauvezin and Neufeld 2015). Furthermore, Tmem9 may interact with ATP6V0D1 indirectly via signal transducer and activator of transcription 3 (STAT3) signaling, leading to lysosomal membrane permeabilization (Chen et al. 2023), because Tmem9 was confirmed to activate STAT3 (Wang, Zhao, et al. 2024), and the ATP6V0D1‐STAT3 complex was reported to regulate lysosomal acidification and lysosomal homeostasis (Chen et al. 2023).

An acidic environment was reported to promote microglia phagocytosis and enlarged plaques through A*β* accumulation (Hu et al. 2009) and faulty lysosomal acidification in microglia impairs phagocytic and autophagic activities, promoting the progression of neurodegenerative disease (Hu et al. 2022). Consistently, we found that the number of A*β* plaques was decreased by PE and RNAi‐Tmem9‐AAV treatments, and this was increased by microglial Tmem9 overexpression. Lysosomal over‐acidification was also reported to impair the activity of lysosomal hydrolases, resulting in aggregation of these neurotoxic proteins (Hu et al. 2022). These contradictory results may be due to the limited degradation ability of A*β* in microglia (Huang et al. 2021). During the early stages, microglial lysosomes have the ability to degrade A*β*. However, disease progression is associated with decreased degradation ability, microglia cell death, abnormal phagocytosis of neurons by microglia, and mediated neuroinflammation, with adverse consequences. Furthermore, aberrant endosomal hyperacidification could also block the ability to recycle multiple cell surface receptors, including A*β* receptors, promoting the development of amyloid plaques by accelerating A*β* production, while inhibiting A*β* clearance or increasing lipid droplet accumulation (Gedam et al. 2023; Prasad and Rao 2018). Consistently, we found that phagocytosis of A*β* in microglial induced lysosomal acidification, in an attempt to degrade toxic material, which was promoted by co‐incubation with LPS. However, LPS treatment enlarged A*β* volumes in BV2 cells, indicating that hyperacidification promoted the growth of A*β* plaques.

We found that ATP6V0D1‐positive vesicles in microglia were leaked and distributed outside the cells, resulting in neuronal loss in these areas. It is reasonable to believe that this lysosomal accumulation contributed to AD pathologies, which is similar to several lysosomal storage disorders, such as Niemann–Pick type C (Lee et al. 2022). Cathepsin exposure after lysosomal leakage to the outside of cells may also accelerate synaptic damage and cell death (Lee et al. 2022). It is noteworthy that overexpression of microglial Tmem9‐ATP6V0D1 signaling may affect other microglial signaling pathways, contributing to the AD pathologies. Firstly, Tmem9 modulates the Wnt/β‐catenin pathway, which is important for microglial synaptic pruning (Wei et al. 2018; Lu et al. 2021). Secondly, TMEM9 regulates the activation of alternative autophagy through interaction with Beclin1, and overwhelmed or dysfunctional autophagy may drive cell death in the AD brain (Baek et al. 2024; Thal, Gawor, and Moonen 2024). Finally, lysosomal acidification regulated by ATP6V0D1 may mediate the mitochondrial‐lysosomal crosstalk, thereby affecting mitochondrial homeostasis and cell survival (Zhou et al. 2022). A limitation of this study is that we only carried out 3‐month exercise intervention to assess the PE‐related protection, leaving the long‐term effects of the treatment unclear. A follow‐up longitudinal study ought to be conducted to evaluate whether the cognitive and synaptic improvements can be sustained over a longer period in future studies.

In summary, our results demonstrated that PE mitigated synaptic loss and protected against cognitive and emotional impairments by down‐regulating microglial Tmem9 expression. Specifically, Tmem9 induced microglial over‐acidification and lysosomal dyshomeostasis through its interaction with ATP6V0D1, resulting in an increase in synapses tagged by C1q and ultimately promoting microglial phagocytosis of these synapses. Therefore, microglial Tmem9 may represent a promising therapeutic target for inhibiting synaptic loss in Alzheimer's disease.

## Author Contributions

Shiyin Li, Mingyue Li, and Ge Li contributed equally to this work. Shiyin Li performed the cell experiments; Ge Li bred the 5xFAD mice and performed behavioral tests; Mingyue Li and Xiaofei He performed the histological staining and western blotting; Xiaofeng Yang and Lili Li performed the two‐photon imaging; Xiaofei He and Shiyin Li drafted the original manuscript; Zejie Zuo, Liying Zhang, and Xiquan Hu revised the manuscript. Xiaofei He and Xiquan Hu conceived and designed the research and edited and revised the manuscript. All authors read and approved the final manuscript.

## Ethics Statement

This study was approved by Guangdong Laboratory Animal Monitoring Institute (IACUC2024102), Guangzhou, China.

## Conflicts of Interest

The authors declare no conflicts of interest.

## Supporting information


Data S1.

## Data Availability

The data that support the findings of this study are available from the corresponding author upon reasonable request.
