# Supplementary material for: Physical Exercise Decreases Complement‐Mediated Synaptic Loss and Protects Against Cognitive Impairment by Inhibiting Microglial Tmem9‐ATP6V0D1 in Alzheimer's Disease
Source: Aging Cell. 2025 Jan 27;24(5):e14496. doi: 10.1111/acel.14496 (PMC12073899; doi:10.1111/acel.14496)
Supplement: Supplementary file 1 — Data S1. [file ACEL-24-e14496-s001.docx]

**Supplementary Materials for**

**Physical exercise decreases complement-mediated synapse loss and protects against cognitive impairment by inhibiting microglial Tmem9-ATP6V0D1 in Alzheimer’s disease**

Shiyin Li^1#^, Mingyue Li^1#^, Ge Li^2#^, Lili Li^1^, Xiaofeng Yang^1^, Zejie Zuo^1^, Liying Zhang^1^, Xiquan Hu^1*^, Xiaofei He^1*^

^#^ **These authors contributed equally to this work.**

^*^ Correspondence to: Xiquan Hu, M.D., Ph.D.,

Department of Rehabilitation Medicine, The Third Affiliated Hospital, Sun Yat-sen University, 600 Tianhe Road, Guangzhou 510630, Guangdong, China.

E-mail: huxiquan@mail.sysu.edu.cn

^*^ Correspondence to: Xiaofei He, M.D., Ph.D.,

Department of Rehabilitation Medicine, The Third Affiliated Hospital, Sun Yat-sen University, 600 Tianhe Road, Guangzhou 510630, Guangdong, China.

E-mail: hexf33@mail.sysu.edu.cn

**Supplementary Materials and Methods**

## Animals and treatments

This study was approved by the Institutional Animal Care and Use Committee (IACUC) of Guangdong Laboratory Animal Monitoring Institute. The 5xFAD mice from the Jackson laboratory (Catalog NO. #034848) were bred in the Guangdong Laboratory Animal Monitoring Institute. Negative mice were taken as wild type (WT), and five-month-old male mice (25-30g) were used in this study. The mice were housed under a 12:12 h light: dark cycle (light on from 07:00−19:00 h), with controlled temperature and humidity and food and water provided ad libitum. 5xFAD male mice were randomly divided into control and physical exercise (PE), RNAi-Tmem9-AAV and PE+OE-Tmem9-AAV groups. Mice in control and RNAi-Tmem9-AAV groups were bred in a common polypropylene (36 cm L × 20 cm W × 14 cm H) cage, whereas mice in PE and PE+OE-Tmem9-AAV groups were bred in a polypropylene cage of the same size, with a 16 cm diameter running wheel, which can rotate when a mouse climbs onto the wheel. The mice were housed under these conditions for three months. All animal experimental procedures followed rules dictated by the animal ethics Committee and was designed in compliance with the ARRIVE guidelines and no exclusion of data was done.

## Proteomic analysis

Most patients with AD are characterized by progressive neurodegeneration in wide areas of the cerebral cortex and the hippocampus (Masters et al., 2015), which are both vulnerable in AD (Hurst et al., 2023), we therefore collected the right brain tissues, including the cortex and hippocampus, to perform proteomic analysis. Specifically, the mice in control-5xFAD and PE-5xFAD groups (n=3 per group) were sacrificed after three months of physical exercise. Then the brain tissues were collected and non-labeled quantitative proteomics sequencing analysis (Label-free) was performed (Figure 1A) (Shanghai Genechem Co.,Ltd), to identify the regulation of exercise on the differential gene expression in AD mice brain. Specifically, the SDT buffer (4%SDS, 100mM Tris-HCl, pH 7.6) were added into the brain tissue, and then the tissues were homogenized. After sonication and centrifugation, supernatant liquids were collected and filtrated, the protein concentration was quantified with the BCA Protein Assay Kit (P0012, Beyotime). For SDS-PAGE electrophoresis, mixing proteins for each sample with loading buffer (P0015F, Beyotime) respectively and boiled for 5 min. The proteins were separated on SDS-PAGE gel. Protein bands were visualized by Coomassie Blue R-250 staining. For FASP (filter-aidedsample preparation) enzymolysis, the protein was reduced with DTT (43819-5G, Sigma). Then the detergent, DTT and other low-molecular-weight components were removed using UA buffer (8M Urea, 150mM Tris-HCl, pH 8.5) by repeated ultrafiltration. Then iodoacetamide (I1149-5G, Sigma) was added to block reduced cysteine residues and the samples were incubated for 30 min in darkness. The filters were washed with UA buffer and NH4HCO3 (A6141-25G, Sigma) buffer. Finally, the protein suspensions were digested with trypsin (V5117, Promega) overnight at 37 °C, and the resulting peptides were collected as a filtrate. The peptide segment was desalted by C18 column (IonOpticks, Australia). The peptide content was estimated by UV light spectral density at 280 nm. LC-MS/MS analysis was performed on a Orbitrap Exploris 480 mass spectrometer (Thermo Fisher Scientific, USA) that was coupled to Easy nLC (Thermo Fisher Scientific, USA). Samples were analyzed by mass spectrometry using a Orbitrap Exploris 480 mass spectrometer. The MS data were analyzed using MaxQuant software version 1.6.17.0. The cutoff of global false discovery rate (FDR) for peptide and protein identification was set to 0.01. Protein abundance was calculated on the basis of the normalized spectral protein intensity (LFQ intensity). Proteins which Fold change > 1.2 and *P* value (Student’s t test) < 0.05 were considered to be a differentially expressed protein.

The process of GO annotation of target protein collections using Blast2GO (V1.3.3) can be roughly summarized into four steps: sequence alignment (Blast), GO terms extraction (Mapping), GO annotation (Annotation) and supplementary annotation (ANNEX). During the KEGG pathway annotation process on the target protein sequences, the KOALA (KEGG Orthology And Links Annotation, V2.2) software was used to align the KEGG GENES database (version: KO_INFO_END.txt (2022.01.25) with the target protein sequences. Based on the Fisher's Exact Test, we compared the distribution of each GO term or KEGG pathway in the target protein set and the total protein set. Then, we could evaluate the significance level of the enrichment of a GO term or KEGG pathway in this way.

## Intracerebroventricular (i.c.v) injection of AAV

To explore the regulation of microglial Tmem9 in AD pathologies, we intracerebroventricularly injected the pAAV-F4/80p-EGFP-mir155siRNA (Tmem9)-SV40 PolyA (2.03 × 10^13^ genomic copies mL^−1^) (GeneChem, China), which was defined as RNAi-Tmem9 group, to knockdown the microglial Tmem9 under control of the F4/80 promoter. Besides, to explore the involvement of Tmem9 down-regulation in PE associated protection, we intracerebroventricularly injected pAAV-F4/80p-MCS-EGFP-3Flag-SV40 PolyA (4.99 × 10^13^ genomic copies mL^−1^) (GeneChem, China) to overexpress the microglial Tmem9 in the PE-5xFAD mice, which was defined as the PE+OE-Tmem9 group (Figure 2A). Specifically, the mice were anesthetized using 4% isoflurane (0.8 L/min) to induce and 2% isoflurane (0.4 L/min), they were then placed on the stereotaxic apparatus (RWD Life Science, China), the right lateral ventricle was orientated as following parameters: posterior: 0.5 mm; mediolateral: 0.5 mm; and dorsoventral: 3 mm. A microsyringe containing 5μL AAV liquid was slowly pumped into the lateral ventricles at 0.5 μL/min with a mini-pump controller (RWD Life Science, China). Ten minutes after injection, the microsyringe was drawn out and the mouse scalp was sutured. The over-expression and down-regulation of microglial Tmem9 were confirmed by western blots and immunofluorescent staining.

## Open field test

Anxiety and exploratory activity were detected by open field test (Seibenhener & Wooten, 2015). Specifically, the apparatus was consisted of a 50 x 50 cm square arena with 40 cm high walls. A video camera was set up directly above, with a field of view that covered the entire interior of the open field. The mice were placed in the center of the field, and spontaneous locomotor activity over a 5-minute period test was recorded, the trajectories were tracked by the video camera, and the time spent in the center (Region of interest, ROI) were recorded using animal behavior analysis software (Xinxin Technology Co., LTD., China).

## Novel Object Recognition (NOR)

Following the open field test, the NOR was carried out to test the mice’s exploratory behavior of novelty. In detail, the mice were accustomed to a blue square chamber (approx. 40 cm × 40 cm × 40 cm) with a white floor (manufactured by Xinxin Technology Co.,LTD., China). On the training day, the mice were placed in the open arena and exposed to two identical familiar objects (square blue blocks in 3 cm diameter) for 10 min. With one in the southeast corner and the other in the southwest corner, each object was bound to the floor. On the test day, the object in the southeast corner was replaced by a novel object (a red cylinder block with a diameter of 3 cm). The mice were allowed to explore each of the distinct objects (familiar and novel) for 5 min. The trajectory of each mouse was monitored using a camera positioned accurately above the test area. The cameras automatically recorded the time spent in the center. The percentage time spent with each object was calculated. Finally, a recognition or preference index could be computed as described in a previous study (Antunes & Biala, 2012), which was determined by the percentage of total object exploration time spent exploring the novel object.

## Morris water maze

Morris water maze was performed to test the spatial learning and memory in mice as described previously (Vorhees & Williams, 2006). Briefly, the apparatus was consisted of a cylindrical tub with an overhead video camera and the automated software (Xinxin Technology Co., LTD., China). The tub had a diameter of 120 cm and a height of 50 cm. The tub was divided into four quadrants, with the endpoints of the tub wall as mice entry points, and a white circular platform with 10-cm diameter was placed 1 cm below the water surface in the center of one of the quadrants. During water maze training, mice were placed into the water facing the tub wall for four quadrant trials per day for five consecutive days. The swim paths and the time to find the platform, referred as escape latency which is up to 60 s were recorded. Mice that failed to reach the platform within 60 s were guided to the platform and kept on the platform for 10 s. The analysis system recorded the animal swimming trajectory data for indices extraction and analysis. On day 6, the platform was removed and a 60-s probe trial was conducted, their swimming speed, the number of times they crossed the target area (former platform) were recorded and analyzed.

## Immunofluorescent staining

The mice were deeply anesthetized with sodium pentobarbital (P-010, Macklin) at concentration of 1% and then transcardially perfused with 0.9% physiological saline (HH02001-1, Biofiven) and 4% (w/v) paraformaldehyde (PFA) (DF0135-500, Biosharp). Brains were removed and fixed in 4% PFA overnight, then gradientally dehydrated by 20% and 30% sucrose (SJ-T6-01-01DM, Damao) until completely precipitated. Subsequently, brain samples were embedded with optimal cutting temperature compound (OCT) (4583, Sakura) and serial 10 µm coronal sections were sectioned using a frozen microtome (Leica CM 1950; Leica Biosystem, Heidelberg, Germany) and stored at -80℃ for later experiments. Sections were microwaved in citrate buffer (pH 6.0) (BL619A, Biosharp) for 5 min, and treated with immunol staining blocking buffer (P0102, Beyotime) for 1 h at room temperature after cooled, followed by incubation overnight at 4℃ with a primary antibody (1:400 anti-Iba1 antibody, Catalog No. GB12105, Servicebio, China; 1:400 anti-PSD95 antibody, Catalog No. GB11277-100, Servicebio, China; 1:400 anti-C1q antibody, Catalog No. ab11861, Abcam, USA; 1:400 anti-Grasp55 antibody, Catalog No. GB111702, Servicebio, China; 1:400 anti-Aβ1-42 antibody, Catalog No. 805501, Biolegend, USA; 1:400 anti-Lamp1 antibody, Catalog No. DF4806, Affinity, China; 1:400 anti-ATP6V0D1 antibody, Catalog No. 68506-1-Ig, Proteintech, China) , followed by incubation with secondary antibody. Sections were embedded using DAPI-containing Fluoroshield™ (F6057, Sigma). Images were acquired using a Nikon fluorescence microscope (Nikon, Japan) or a confocal microscope (Leica, DM6000, Germany). The number of positive cells was counted using Leica Application Suite X software, immunofluorescent results were analyzed using ImageJ software (National Institutes of Health, Bethesda, Maryland, USA).

## Western blotting

Total protein was extracted using lysis buffer and quantified using the BCA Protein Assay Kit (P0012, Beyotime). Proteins were added (20 µg/lane) in sodium dodecyl sulfate polyacrylamide gels, separated by electrophoresis at 120 V for 90 min, and transferred to a polyvinylidene difluoride (PVDF) membrane (ISEQ00010, Millipore) at 100 V for 2 h. Then, the membrane was sealed in 5% skimmed milk (1172GR500, Biofroxx) or BSA (TJ-10735108001, Tianjun) for 1 h, followed by incubation at 4 °C overnight with primary antibodies, including rabbit anti-Tmem9 (1: 1000, DF12540, Affinity Biosciences, China), anti-CTSB (1: 1000, AF5189, Affinity Biosciences, China), anti-PSD95 (1: 1000, GB11277-100, Servicebio, China)，anti-C1qa (1: 1000, DF7839, Affinity Biosciences, China)，anti-C1qb (1: 1000, DF7283, Affinity, China)， anti-C3 (1: 1000, PA5-21349, ThermoFisher, USA)，anti-Grasp55 (1: 1000, GB111702, Servicebio, China)，anti-β-Tubulin(1: 1000, AF7011, Affinity, China)，anti-GAPDH(1: 1000, AF7021, Affinity, China)，then was washed by PBS and incubated with the relevant secondary antibodies for 1 h. Finally, protein bands were detected using ECL blotting kit (SQ202L, Epizyme) and chemiluminescence imaging system. Gray scale values of immunnoreactive bands were analyzed using ImageJ software.

## Transmission electron microscope (TEM)

Mice were deeply anesthetized and perfused intracardially with 40 mL of pre-cooled 0.9% NaCl. The cortex was obtained and kept overnight in 2% paraformaldehyde and 2.5% glutaraldehyde (G6257, Sigma) in 0.1 M phosphate buffer solution (PBS) (pH 7.4) (G0002-2L, Servicebio), the samples were then subsequently washed 3 times with 0.1 M PBS (10 min each time) and post-fixed by 1% osmic anhydride (SPI-Pon™ 812, SPI) for 1 h at 4°C. The samples were then washed with 0.1 M PBS (15 min per wash) and dehydrated in a gradient of ethanol solutions (50%, 70%, 80%, and 90% for 10 min; 100% for 10 min 2 times). This was followed by dehydration in acetylacetone (123-54-6, Guangzhou), after which samples were gradually embedded with mixtures of acetylacetone and EPON812 (45345, Macklin) (ratios of 3:1 for 0.5 h, 1:1 for 4 h at room temperature) before being embedded in pure EPON812 overnight. The samples were cured in the oven at 60°C for 48 h, samples were then sectioned using an ultra-thin microtome (Leica UC7). 100 nm - thick ultra-thin sections were prepared and stained with 1% uranyl acetate for 20 min followed by lead citrate for 12 min. Images were acquired using a Tecnai Spirit transmission electron microscope (FEI, USA).

## Golgi staining

Golgi-Cox staining was performed strictly according to the manufacturer's instructions (PK401, FD Rapid GolgiStain™ Kit, MD). Specifically, mice brain was quickly removed, rinsed with PBS, and then immersed in a 1:1 mixture of solution A and solution B (containing mercuric chloride, potassium dichromate, and potassium chromate), replaced with a new impregnating liquid after 6h of immersion, and stored at room temperature (RT) in the dark for 2 weeks. The tissues were then transferred to solution C, immersed for 24h and then replaced with new solution C. The tissues were stored at RT and protected from light for 7 days. The brains were then frozen in isopentane and stored at -80 °C. Frozen brain tissue was prepared into coronal sections of 100 µm thickness on a sliding microtome (Leica) at -22 °C, transferred to gelatin-coated microscope slides (LabScientific) containing solution C, and dried in the dark at RT for 3 days. After rinsing the slides with distilled water, the sections were stained with a mixture of solutions D and E for 10 min, rinsed with distilled water and dehydrated in a series of ethanol, hyalinized in xylene, and covered with a neutral resin. Single-plane images were taken under a light field using a microscope with a 100 X oil objective (BX51, Olympus, Japan), and second- or third-stage dendrites were selected for quantitative analysis using RECONSTRUCT software (http://synapses.clm.utexas.edu), and the number of dendritic spines was calculated by ImageJ software.

## Enzyme linked immunosorbent assay (ELISA) for C1q factor

The C1q concentration in plasma was examined using enzyme linked immunosorbent assay (ELISA) kit according to the manufacturer’s instructions (MM-44729M2, Meimian). Specifically, the whole blood from mice were collected in the anticoagulant-treated tubes containing EDTA, the samples were centrifugated at 4℃ for 20 minutes (3000 rpm/min) the supernatants were collected and transferred into a new tube for ELISA. 10μL samples with 40μL sample diluent were added into the 96-well plates containing purified mouse C1q antibody, they were incubated at 37℃ for 30min. After washing five times, HRP-conjugate reagent was added and incubated for 30min at 37℃. Then, chromogen solution was added and incubated for 10min at 37℃ and stop solution was added to terminate the reaction, absorbance was measured using a microtiter plate reader (BioTek Synergy H1, USA) at a wavelength of 450 nm, the standard curve was obtained and the concentration was calculated according to the standard curve.

## Cell culture and drug treatments

The mouse BV2 microglia cell line was purchased from Shanghai Cell Research Center (Shanghai, China). The cells were grown at 37 °C (a 5% CO_2_ atmosphere) in Dulbecco’s Modified Eagle's Medium (DMEM) (C11995500BT, Gibco) supplemented with 10% (vol/vol) fetal bovine serum (FBS) (10099-141, Gibco) and 1% penicillin/streptomycin (15140122, Gibco). 5μM oA*β* was incubated in the BV2 cells for 24 hours to establish the AD model in vitro (oA*β* group). Besides, these oA*β* - incubated cells were pre - treated with LPS (100ng/mL) for 12 hours (LPS + oA*β* group) to examine the regulation of pro-inflammatory condition on the C1q activation.

To examine the regulation of microglial engulfment of synapse in vitro, we co-cultured BV2 cells with HT22 neurons. Specifically, HT22 neuronal cell line was purchased from Wuhan Pricella Biotechnology Co., Ltd. (Wuhan, China), they were plated at low density to allow uniform synapse development for 24 hours. BV2 microglia cells with oA*β* or LPS + oA*β* pre - treatment were seeded into the plate of HT22 cells, at a ratio of 2:1, these mixed cells were then continued co-cultured for 24 hours, then immunofluorescent staining for C1q and PSD95 was performed.

## A*β*42 oligomer preparation

In brief, the HFIP (hexafluoroisopropanol) (H811027, Macklin) treated A*β*42 peptides (52487, Glbiochem) were resuspended in dimethyl sulfoxide (DMSO) (0219605590, MP) followed by dilution to 500 μM in Ham’s F12 (Pm150813, Procell). After incubation for 24 h at 4 °C, the soluble A*β*42 oligomers (oA*β*) were obtained and centrifuged for 10 min at 14,000g.

## MTT Assay

The MTT [3-(4,5-dimethylthiazoldimethylthiazol-2-yl)-2,5-diphenyl tetrazolium bromide] assay (E-CK-A341, Elabscience) was conducted to determine the cytotoxicity of oA*β* to BV2 cells. BV2 microglia were cultured in a 96-well plate at 5000 cells/well, they were cultured with oA*β* at concentration of 1μM, 5μM, 10μM, 15μM or 20μM for 24 h, 100 μL of 1×MTT working solution per well was added and incubated at 37°C under a humidified atmosphere with 5% CO2 for 2 h., 150 μL DMSO was then added into each well. The plate was shaken for 5 min. Absorbance was then determined using a microplate reader ((BioTek Synergy H1, USA) at 570 nm. The percentage growth inhibition was calculated to determine the cytotoxicity.

## Lentivirus transfection

A negative control (Catalog #: LPP-NEG-Lv201-050), Tmem9-cDNA (Catalog #: LPP-Mm08429-Lv201-100) and Tmem9-ShRNA-containing (Catalog #: LPP-MSH033519-LVRU6GP-d-200, Catalog #: LPP-MSH033519-LVRU6GP-e-200, Catalog #: LPP-MSH033519-LVRU6GP-f-200) lentivirus reagents were obtained from Guangzhou iGenebio Biotechnology Co., Ltd (Guangzhou, China), the sequences are shown in Supplementary Table 4. Mouse BV2 microglia were transfected with these lentiviruses according to the manufacture’s protocol. Specifically, BV2 cells in the logarithmic growth phase were seeded in high-glucose DMEM containing 10% FBS (10099-141, Gibco) in a 6-well plate, with 3 × 10^5^ cells per well, the cells were transfected by replacing the original medium with transfection medium containing 30 μL 1 × 10^8^ TU/mL lentivirus reagents, 5ug/mL Polybrene (LT010-S, GeneCopoeia) and 0% FBS for 12 hours. The transfection medium was then replaced by high glucose DMEM with 10% FBS, and the cells were cultured for another 72 hours. The over-expression or knockdown of Tmem9 were evaluated by expression of green fluorescent protein and real-time Polymerase Chain Reaction (RT-qPCR).

## Measurement of lysosomal pH value

Lysosomal pH value in microglia was measured by LysoView Red (CS343, ZoFtic) according to the manufacturer’s instruction. Briefly, BV2 cells were seeded and cultured overnight in a 24-well plate, with a 9 mm coverslip in each well, the cells were incubated with 1 μM LysoView Red (stock 1 mM in DMSO) at 37 °C for 2h and then washed twice with ice-cold PBS buffer. Cells were fixed in 4%PFA for 15 min, and the coverslips were embedded with DAPI and mounted on the slide. The slices were observed under a confocal microscopy (Leica M165 FC, Heidelberger, Germany), and images were captured at 577nm excitation/590nm emission (red), and the fluorescent pixels were analyzed by ImageJ software. LysoView Red exhibits pH - dependent increase of fluorescence intensity under acidic conditions.

## Statistical analysis

All data were analyzed by an investigator blinded to the group allocation. Image J (National Institutes of Health, USA) was used to analyze the immunofluorescent and western blots results. GraphPad Prism 8.0 was used for conducting statistical analysis. For analysis of the latencies to the platform during water maze training, repeated measures two-way analysis of variance followed by Tukey’s post hoc multiple comparison tests were performed. For other data, one-way analysis of variance (ANOVA) followed by Tukey’s post hoc multiple comparison tests were performed. All data were presented as means ± standard deviations (Mean ± S.D.), and *P* < 0.05 was considered statistically significant.

**Supplementary Figure Legends**


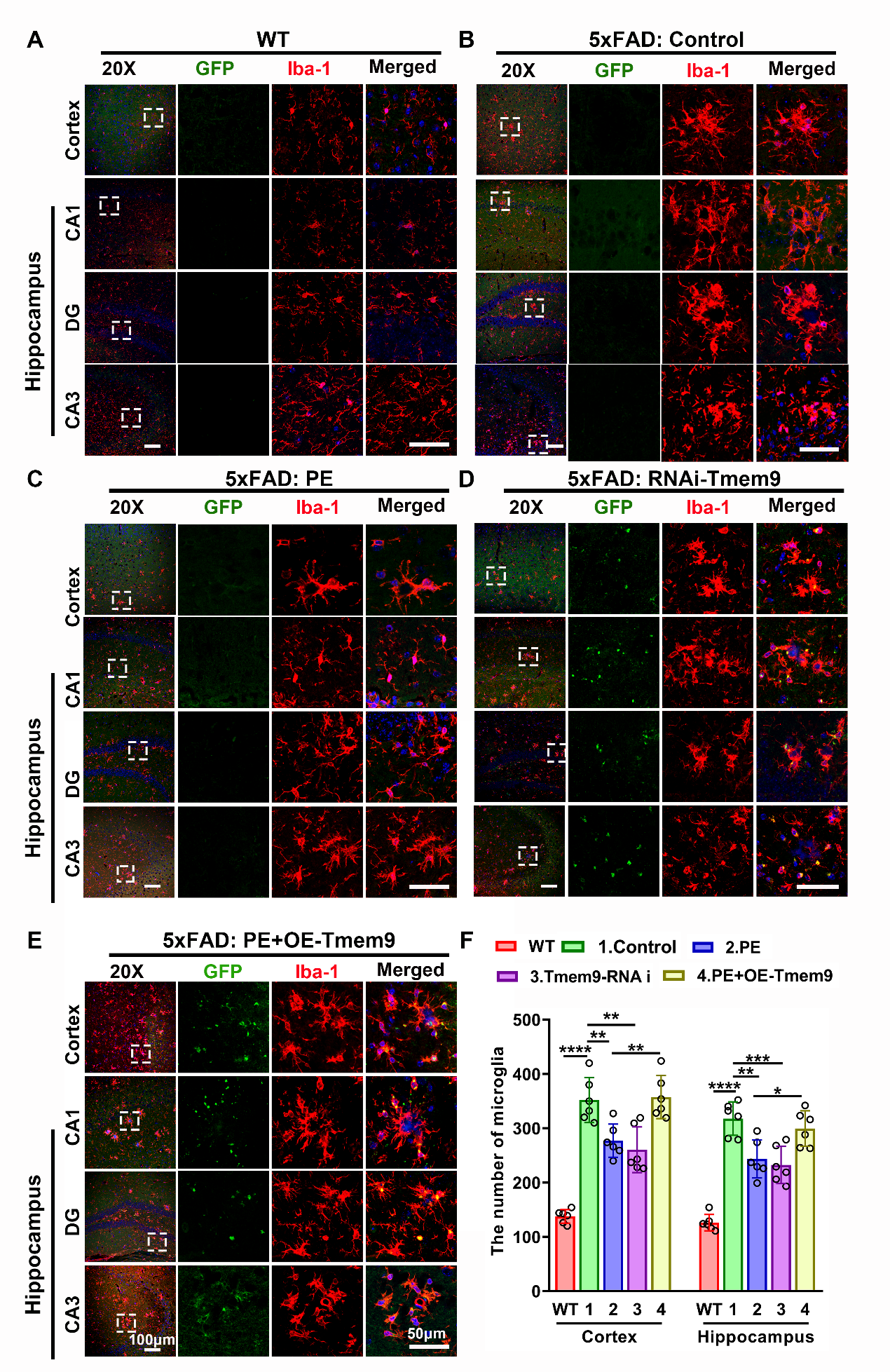


**Figure S1.** **AAV-GFP expressions in the RNAi-Tmem9 and PE + OE-Tmem9 groups were co-localized within Iba-1 microglia.** A) Representative confocal images for the GFP^+^ microglia of cortex and hippocampus in WT group. B) Representative confocal images for the GFP^+^ microglia of cortex and hippocampus in Ctrl-5xFAD group. C) Representative confocal images for the GFP^+^ microglia of cortex and hippocampus in PE-5xFAD group. D) Representative confocal images for the GFP^+^ microglia of cortex and hippocampus in RNAi-Tmem9 5xFAD group. E) Representative confocal images for the GFP^+^ microglia of cortex and hippocampus in PE + OE-Tmem9 5xFAD group. E) Immunofluorescence analysis for the microglia in cortex and hippocampus among the WT, Ctrl-5xFAD, PE-5xFAD, RNAi-Tmem9 5xFAD and PE + OE-Tmem9 5xFAD groups. Scale bar, 20 µm and 100 µm. Dataset expressed as the mean ± S.D. **P* < 0.05; ***P* < 0.01; ****P* < 0.001; *****P* < 0.0001.


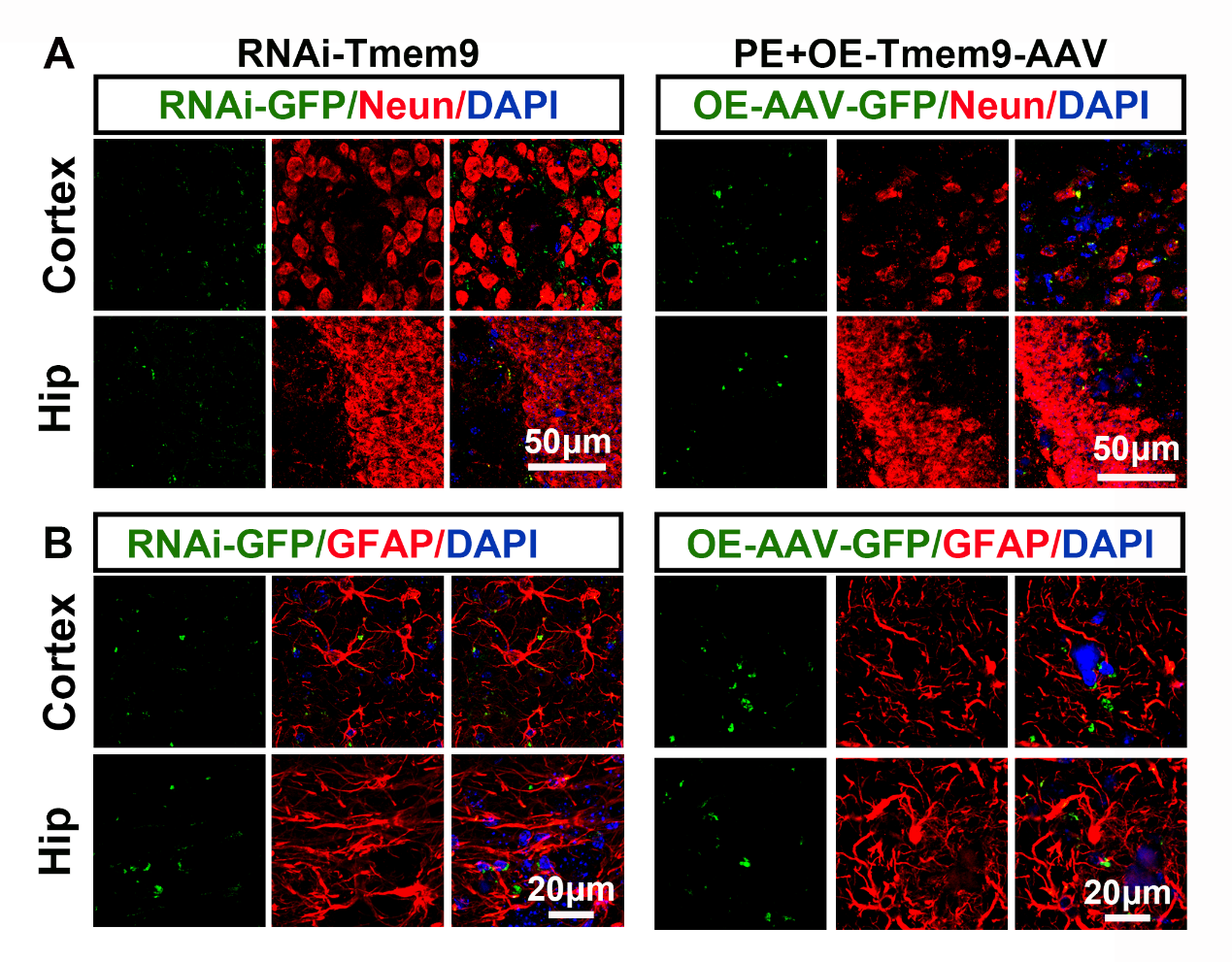


**Figure S2. AAV-GFP expressions in the RNAi-Tmem9 and PE + OE-Tmem9 groups were not co-localized in neurons or astrocytes.** A) Representative confocal images for the GFP^+^ neurons and GFP^+^ astrocytes of cortex and hippocampus in RNAi-Tmem9 5xFAD and PE + OE-Tmem9 5xFAD groups. Scale bar, 50 µm and 20 µm.


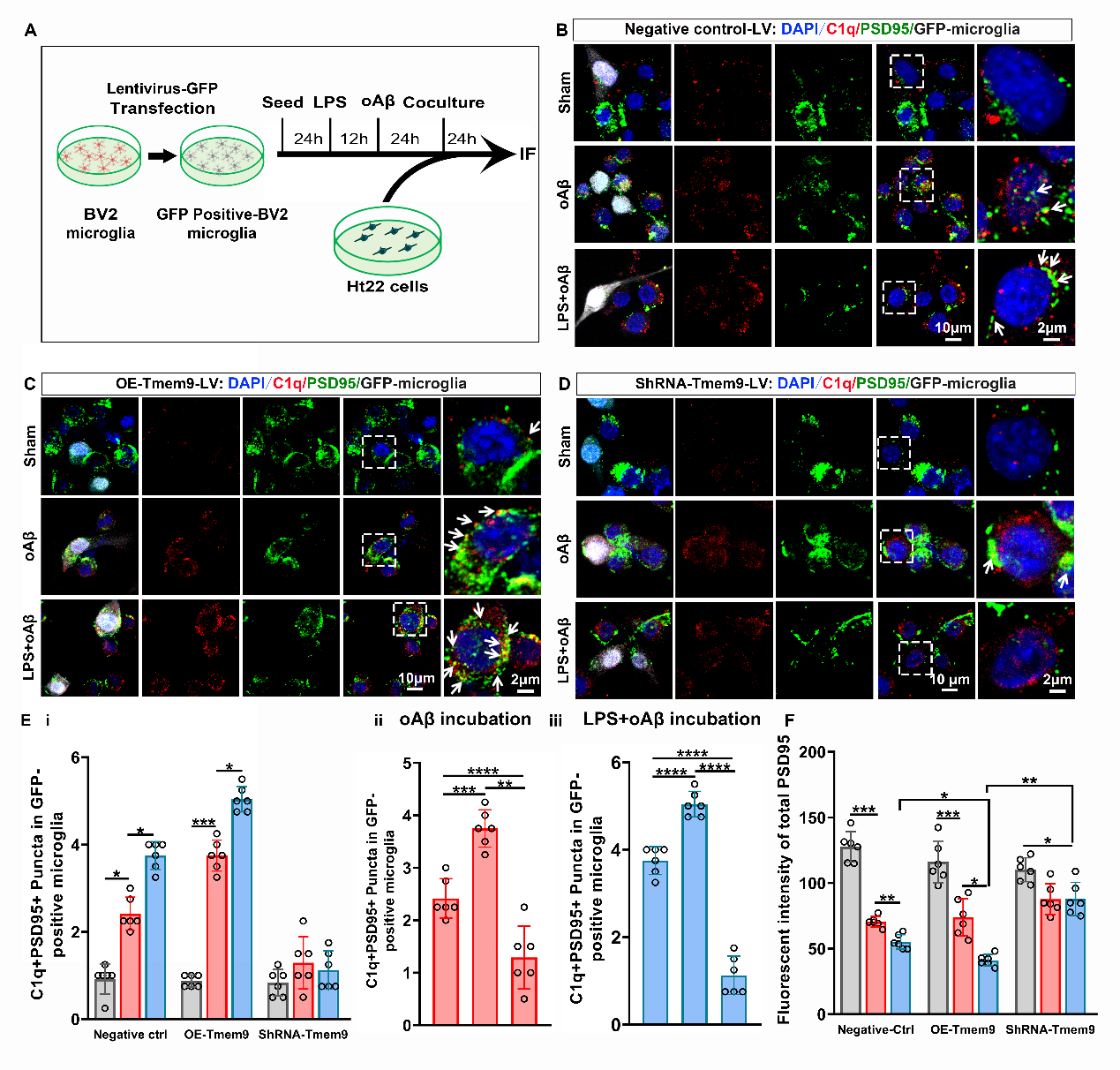


**Figure S3. Tmem9 contributed to C1q-mediated microglial engulfment of synapses *in vitro***. A) Schematic for treatment of BV2 microglia and co-culture with HT22 neuron cells. Drawn by https://gdp.rjmart.cn/. B) Representative confocal images of C1q^+^PSD95^+^ puncta (white arrows) in BV2 cells with negative control-Lv transfection (gray color) among the sham, oA*β* and LPS + oA*β* groups. Left panel: white dashed boxes label the areas that are magnified in the right panel. Right panel: magnified images from the areas in the white dashed box. C) Representative confocal images of C1q^+^PSD95^+^ puncta (white arrows) in BV2 cells with OE-Tmem9-LV transfection (gray color) among the sham and oA*β* and LPS + oA*β* groups. Left panel: white dashed boxes label the areas that are magnified in the right panel. Right panel: magnified images from the areas in the white dashed box. D) Representative confocal images of C1q^+^PSD95^+^ puncta in BV2 cells with ShRNA-Tmem9-LV transfection among the sham, oA*β*, and LPS + oA*β* groups. Left panel: white dashed boxes label the areas that are magnified in the right panel. Right panel: magnified images from the areas in the white dashed box. E) Comparison analysis for the C1q^+^PSD95^+^ puncta among the sham, oA*β*, and LPS + oA*β* groups in BV2 cells with negative control-LV transfection, OE-Tmem9-LV transfection and ShRNA-Tmem9-LV transfections. Dataset expressed as the mean ± S.D. **P* < 0.05; ***P* < 0.01; ****P* < 0.001; *****P* < 0.0001.


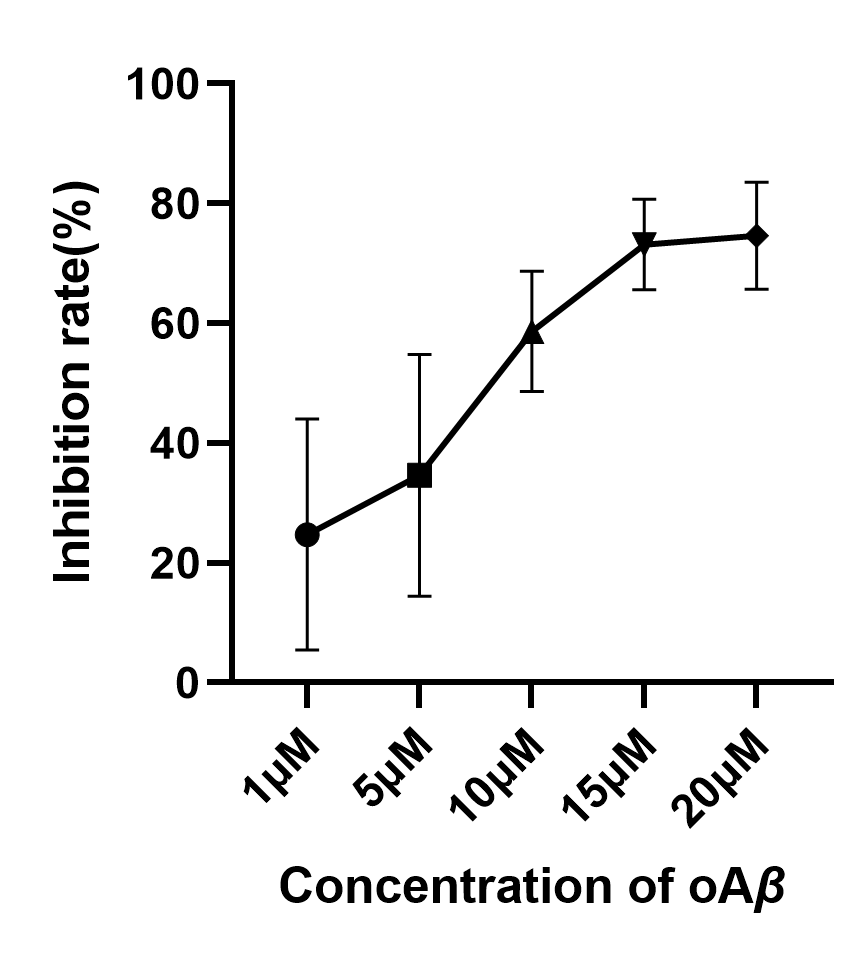


**Figure S4. Inhibition of BV2 cell growth was increased with increasing oA*β* concentrations.** BV2 cell inhibition rate exposed or not exposed to oA*β* (1–20 μM) for 24 hours was assessed by 3-(4,5-dimethylthiazol-2-yl)-2,5-diphenyltetrazolium bromide (MTT) assay. Each MTT value reflects the number of viable cells. Inhibition ratio is shown as the ratio to each MTT value of vehicle treatment.


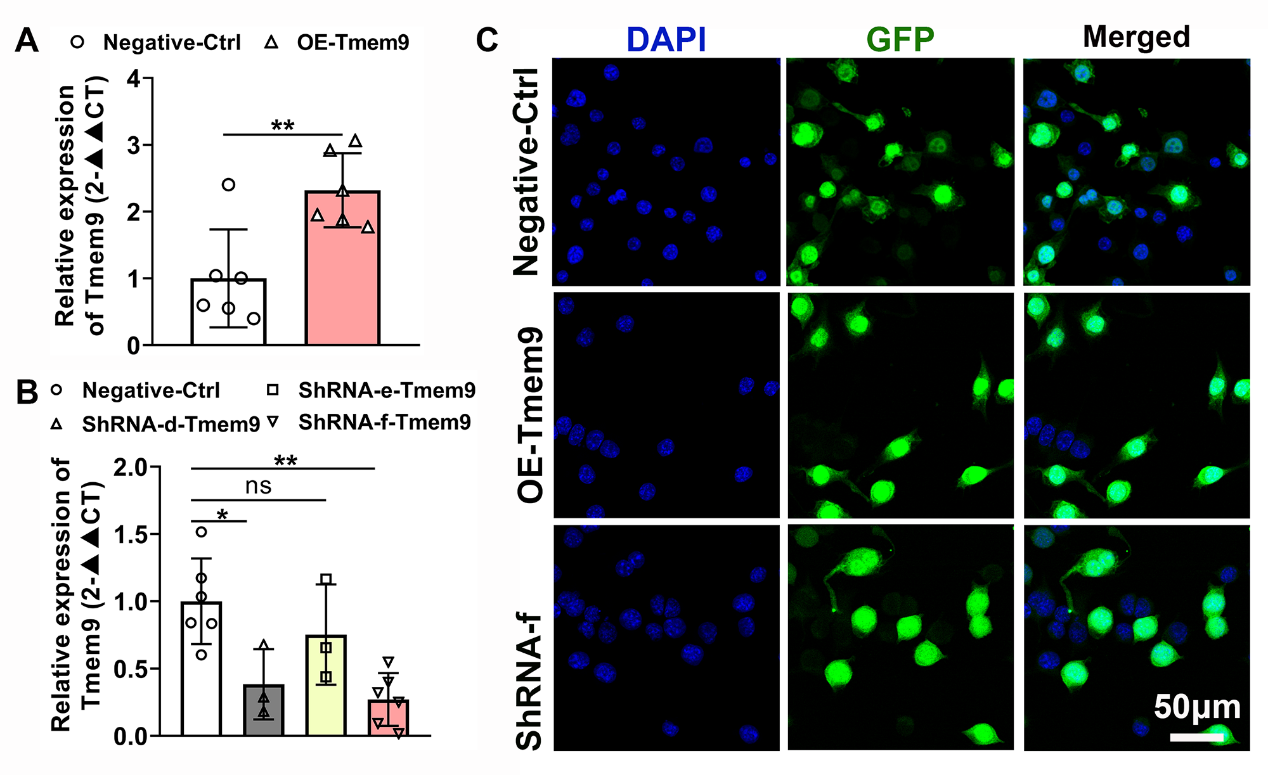


**Figure S5. Overexpression or knockdown of Tmem9 was confirmed by RT-qPCR and immunofluorescent staining.** A) Reverse transcription polymerase chain reaction (RT-PCR) analysis of Tmem9 between BV2 with negative control-LV transfection and BV2 with OE-Tmem9-LV transfection. B) Reverse transcription polymerase chain reaction (RT-PCR) analysis of Tmem9 between BV2 with negative control-LV transfection and BV2 with ShRNA-d-Tmem9-LV transfection, ShRNA-e-Tmem9-LV transfection and ShRNA-f-Tmem9-LV transfection. C) Representative images of GFP expression among BV2 with negative control-LV transfection, OE-Tmem9-LV transfection and ShRNA-Tmem9-LV transfection. Scale bar, 50 μm. Dataset expressed as the mean ± S.D. **P* < 0.05; ***P* < 0.01; ****P* < 0.001; *****P* < 0.0001.


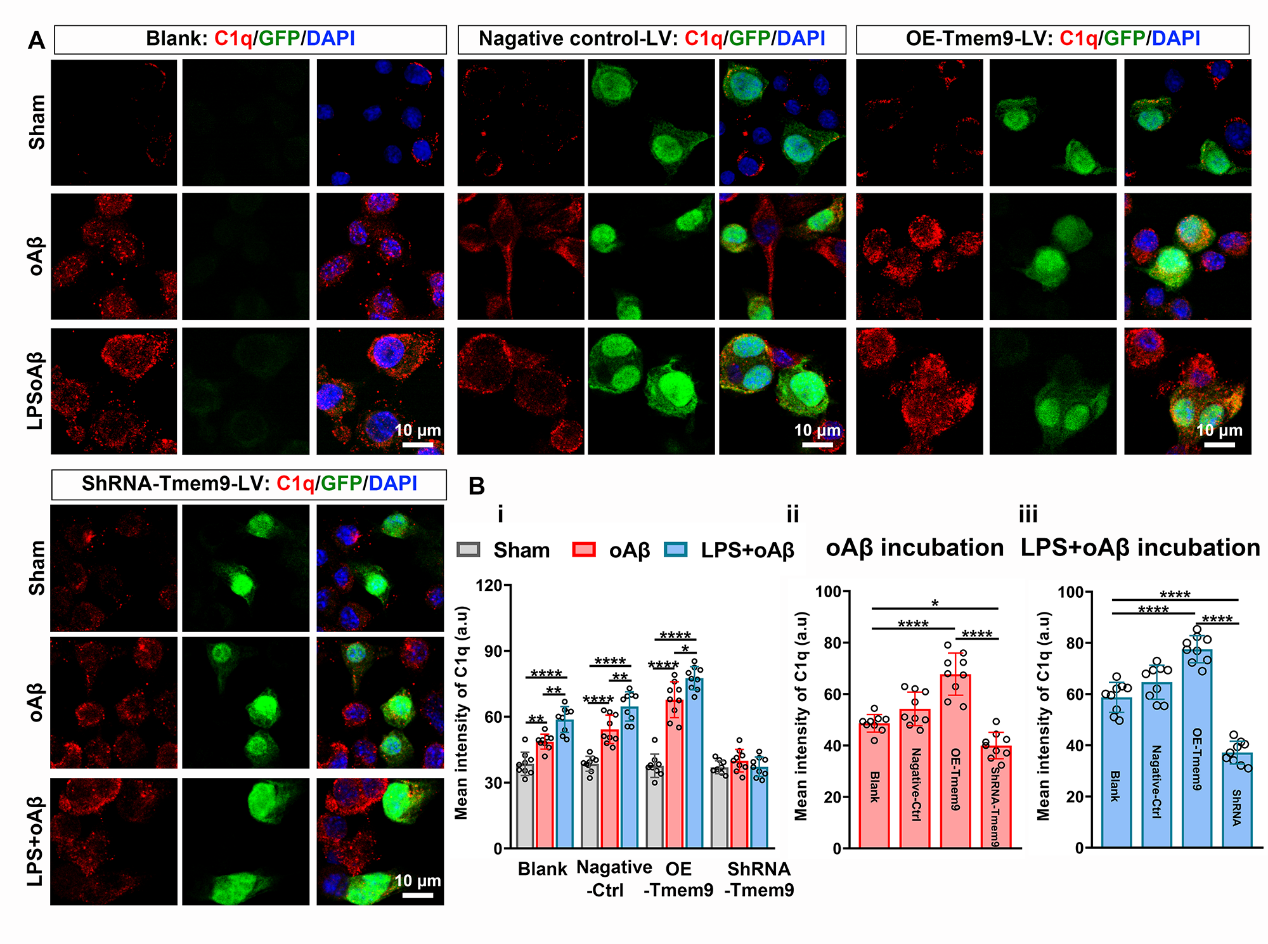


**Figure S6. Knockdown of microglial Tmem9 inhibited the activation of C1q factors, while overexpression of microglial Tmem9 promoted the activation.** A) Representative images of C1q among BV2, BV2 with negative control-LV transfection, OE-Tmem9-LV transfection and ShRNA-Tmem9-LV transfection. Scale bar, 10 μm. B) Immunofluorescence analysis for the C1q among sham, oA*β* and LPS + oA*β* group (i), and immunofluorescence analysis for the C1q among BV2, BV2 with negative control-LV transfection, OE-Tmem9-LV transfection, ShRNA-Tmem9-LV transfection incubating with oA*β* (ii) or LPS + oA*β* (iii). Dataset expressed as the mean ± S.D. **P* < 0.05; ***P* < 0.01; ***P < 0.001; ****P < 0.0001.


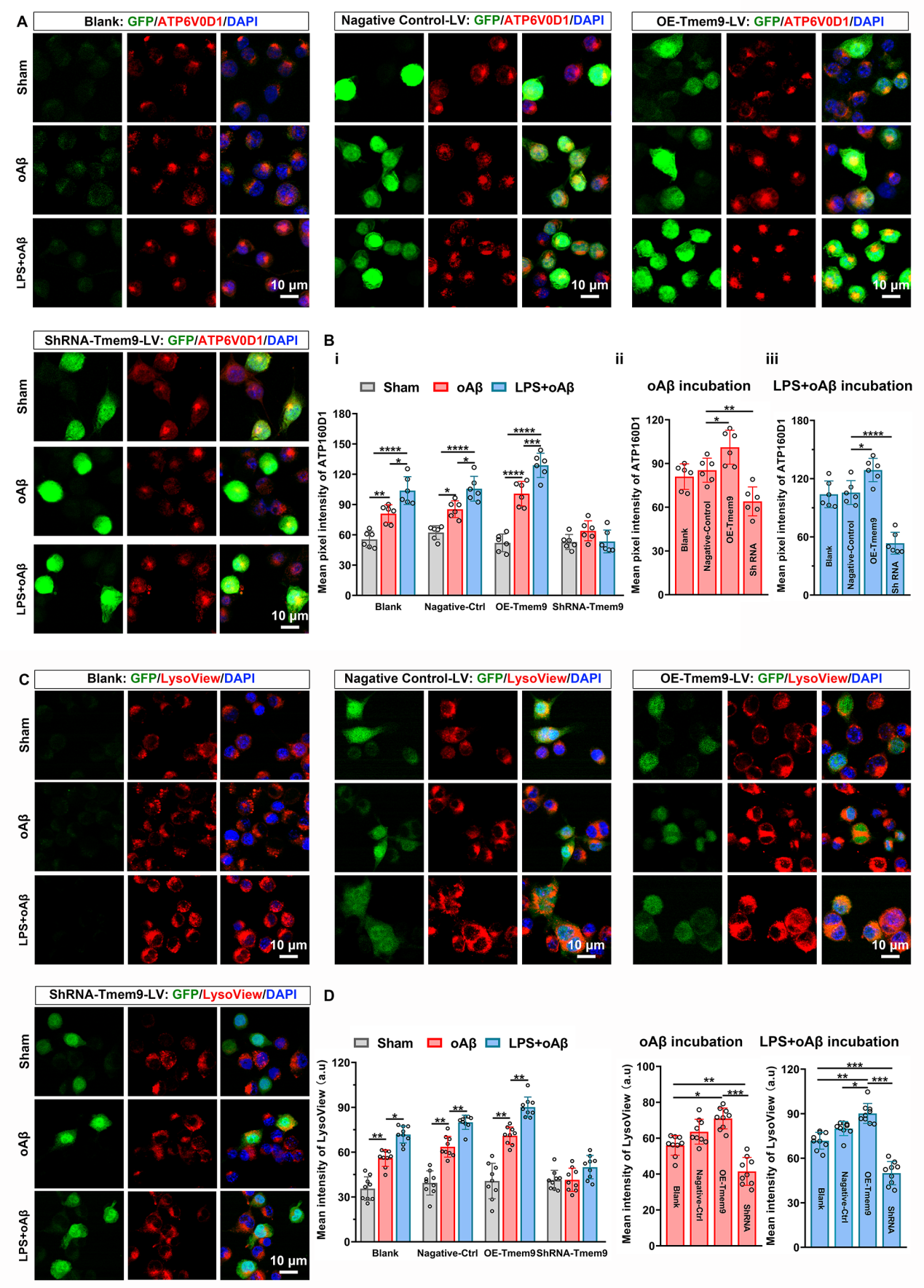


**Figure S7. Overexpression of Tmem9 was enhanced, while knockdown of Tmem9 decreased ATP6V0D1 expression, to regulate lysosomal acidification in microglia.** A-B) Immunofluorescent analysis for ATP6V0D1 expressions in BV2 cells with negative control-LV transfection, OE-Tmem9-LV transfection, and ShRNA-Tmem9-LV transfection among the sham, oA*β* and LPS + oA*β* groups. C-D) Immunofluorescent analysis for LysoView Red intensities, an indicator for lysosomal pH values, in BV2 cells with negative control-LV, OE-Tmem9-LV, and ShRNA-Tmem9-LV transfections among the sham, and oA*β* and LPS + oA*β* groups. The results are expressed as the mean ± S.D. **P* < 0.05; ***P* < 0.01; ****P* < 0.001; *****P* < 0.0001.


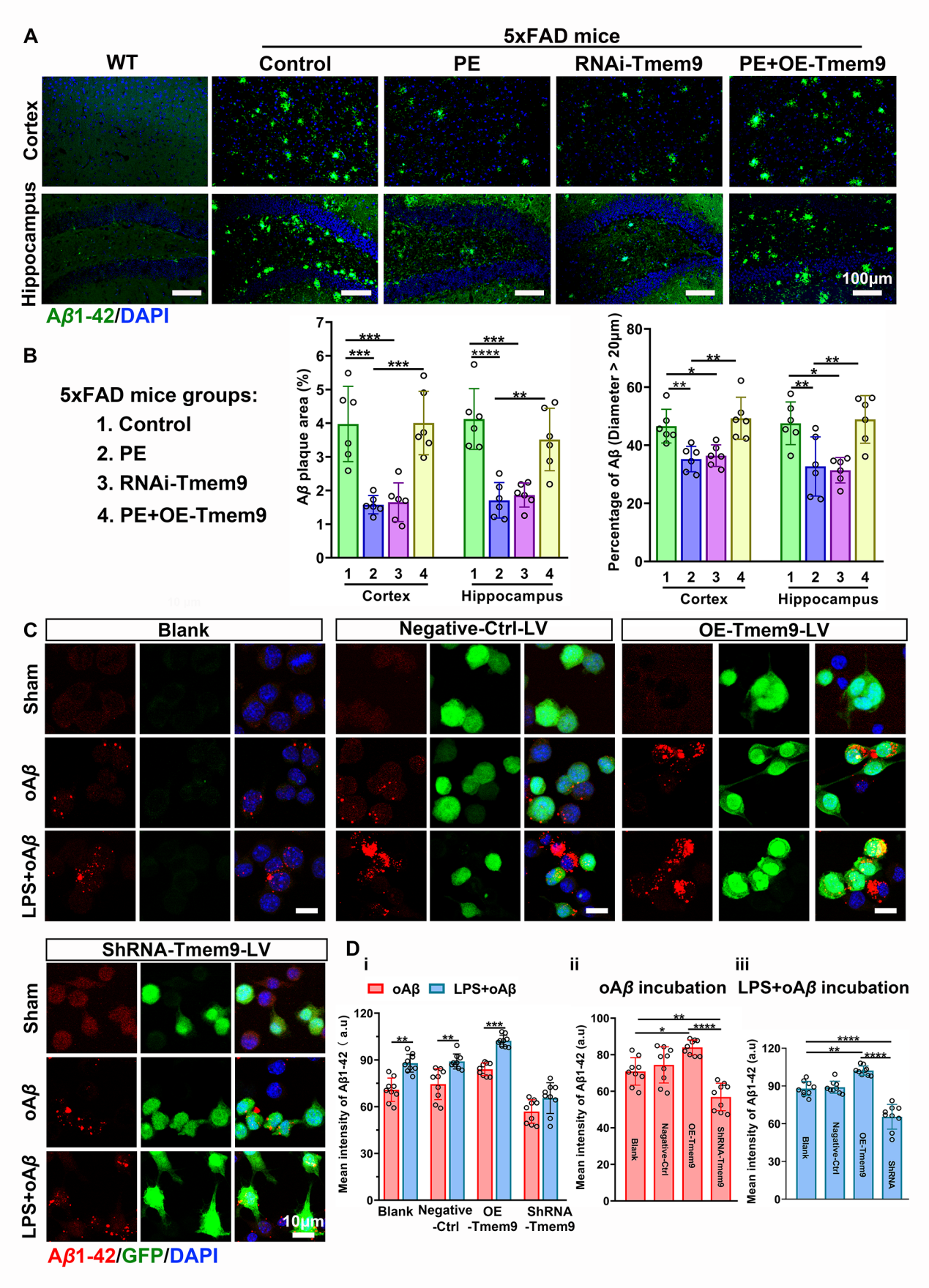


**Figure S8. Downregulation of microglial Tmem9 decreased amyloid beta deposition both *in vitro* and *in* *vivo,* which was related to the PE-mediated protection.** A-B) Immunofluorescence analysis of the amyloid beta in microglia among the WT, Ctrl-5xFAD, PE-5xFAD, RNAi-Tmem9, and PE + OE-Tmem9 groups. C-D) Immunofluorescent analysis of A*β*1-42 in BV2 cells with negative control-Lv transfection, OE-Tmem9-LV transfection, ShRNA-Tmem9-LV transfection among sham, oA*β,* and LPS + oA*β* groups. The results are expressed as the mean ± S.D. ^*^*P* < 0.05; ^**^*P* < 0.01; ^***^*P* < 0.001; ^****^*P* < 0.0001.

**Table S1 The forward and reverse primers used in this study**

| **Gene** | **Forward Primer** | **Reverse Primer** |
| --- | --- | --- |
| Tmem9 | GTCCGCCTTACAGAAACATCA | CTCGTACCTACACTCGCAGAG |
| β-actin | GTGACGTTGACATCCGTAAAGA | GCCGGACTCATCGTACTCC |

**Table S2 Key resources table**

| **Reagent or Resource** | **Source** | **Identifier** |
| --- | --- | --- |
| **Antibodies** | | |
| anti-ATP6V0D1 | Proteintech | 68506-1-Ig |
| anti-Lamp1 | Affinity Biosciences | DF4806 |
| anti-Aβ1-42 | Biolegend | 805501 |
| anti-C1q | Abcam | ab11861 |
| anti-Iba1 | Servicebio | GB12105 |
| anti-GAPDH | Affinity Biosciences | AF7021 |
| anti-β-Tubulin | Affinity Biosciences | AF7011 |
| anti-Grasp55 | Servicebio | GB111702 |
| anti-C3 | Thermo Fisher Scientific | PA5-21349 |
| anti-C1qb | Affinity Biosciences | DF7283 |
| anti-C1qa | Affinity Biosciences | DF7839 |
| anti-PSD95 | Servicebio | GB11277-100 |
| anti-CTSB | Affinity Biosciences | AF5189 |
| anti-Tmem9 | Affinity Biosciences | DF12540 |
| Anti-rabbit lgG, HRP-linked Antibody | Cell Signaling Technology | 70745 |
| Anti-mouse IgG, HRP-linked Antibody | Cell Signaling Technology | 7076S |
| Anti-mouse IgG (H+L), F(ab')2 Fragment (Alexa Fluor® 647 Conjugate) #4410 | Cell Signaling Technology | 4410S |
| Anti-mouse IgG (H+L), F(ab')2 Fragment (Alexa Fluor® 488 Conjugate) | Cell Signaling Technology | 4408S |
| Anti-rabbit IgG (H+L), F(ab')2 Fragment (Alexa Fluor® 555 Conjugate) | Cell Signaling Technology | 4413S |
| **Chemical, peptides, and recombinant proteins** | | |
| Urea | BIO-RAD | 161-0731 |
| SDS | Sangon | SB0485-500g |
| Tris | Sangon | T0826-500g |
| Iodoacetamide (IAA) | Sigma | I1149-5G |
| NH4HCO3 | Sigma | A6141-25G |
| Formic acid | Thermo Fisher Scientific | A117 |
| Acetonitrile | Merck | 1000304008 |
| SDS-PAGE loading buffer | Beyotime | P0015F |
| C18 Cartridge | Waters | WAT023590 |
| Trypsin | Promega | V5117 |
| HCl | Sinopharm | 10011018 |
| DTT | Sigma | 43819-5G |
| SDS-PAGE gel | Epizyme | PG113 |
| isoflurane | RWD | R510-22-10 |
| sodium pentobarbital | Macklin | P-010 |
| 0.9% physiological saline | BIOFIVEN | HH02001-1 |
| 4% (w/v) paraformaldehyde (PFA) | Biosharp | DF0135-500 |
| sucrose | Damao | SJ-T6-01-01DM |
| optimal cutting temperature compound (OCT) | SAKURA | 4583 |
| citrate buffer (pH 6.0) | biosharp | BL619A |
| Immunol Staining Blocking Buffer | Beyotime | P0102 |
| DAPI-containing Fluoroshield™ | Sigma | F6057 |
| polyvinylidene difluoride (PVDF) membrane | Millipore | ISEQ00010 |
| skim milk powder | Biofroxx | 1172GR500 |
| PBS | Servicebio | G0002-2L |
| 1% osmic anhydride | SPI | SPI-Pon™ 812 |
| Acetylacetone | Guangzhou | 123-54-6 |
| EPON812 | Macklin | 45345 |
| 1% uranyl acetate | SPI | SPI-02624 |
| 2.5% glutaraldehyde | Sigma | G6257 |
| Dulbecco’s Modified Eagle's Medium (DMEM) | Gibco | C11995500BT |
| fetal bovine serum (FBS) | Gibco | 10099-141 |
| 1% penicillin/ streptomycin | Gibco | 15140122 |
| LPS (100ng/mL) | TargetMol | T11855-1mg |
| HFIP (hexafluoroisopropanol) | Macklin | H811027 |
| Aβ42 peptides | Glbiochem | 52487 |
| dimethyl sulfoxide (DMSO) | MP | 0219605590 |
| Ham’s F12 | Procell | Pm150813 |
| Polybrene | GeneCopoeia | LT010-S |
| BSA | Tianjun | TJ-10735108001 |
| **Virus** |  |  |
| pAAV-F4/80p-EGFP-mir155siRNA (Tmem9)-SV40 PolyA | GeneChem | Tmem9-RNAi (128836-1) |
| pAAV-F4/80p-MCS-EGFP-3Flag-SV40 PolyA | GeneChem | AAV9-Tmem9 (99985-2) |
| Lentiviral particles for Negative Control | iGenebio Biotechnology | LPP-NEG-Lv201-050 |
| Lentiviral particles for Tmem9 | iGenebio Biotechnology | LPP-MSH033519-LVRU6GP-f-200 |
| Lentiviral particles for Tmem9 | iGenebio Biotechnology | LPP-MSH033519-LVRU6GP-e-200 |
| Lentiviral particles for Tmem9 | iGenebio Biotechnology | LPP-MSH033519-LVRU6GP-d-200 |
| Lentiviral particles for Tmem9 | iGenebio Biotechnology | LPP-Mm08429-Lv201-100 |
| Lentiviral particles for Scrambled Control | iGenebio Biotechnology | LPP-CSHCTR001-LVRU6GP-200 |
| **Materials** |  |  |
| C18 column | IonOpticks | AUR3-25075C18-TS |
| 30kD ultrafiltration tube | Sartorius | VN01H22 |
| 0.22 μm ultrafiltration tube | Corning Spin-X | 8160 |
| Multiple Affinity Removal LC Column – Mouse 3 | Agilent | 5188-5218 |
| **Critical commercial assays** | | |
| C1q ELISA kit | Meimian | M-44729M2 |
| BCA Protein Assay Kit | Beyotime | P0012 |
| ECL blotting kit | Epizyme | SQ202L |
| FD Rapid GolgiStain™ Kit, MD | FD NeuroTechnologies | PK401 |
| MTT assay | Elabscience | E-CK-A341 |
| LysoView Red | ZoFtic | CS343 |
| **Experimental models: Organisms/strains** | | |
| 5xFAD mice | Jackson laboratory | Catalog NO. #034848 |
| The mouse BV2 microglia cell line | Shanghai Cell Research Center | BFN608006363 |
| The mouse HT22 neuronal cell line | Wuhan Pricella Biotechnology | Cl-0697 |

**References**

Antunes, M., & Biala, G. (2012). The novel object recognition memory: neurobiology, test procedure, and its modifications. *Cognitive processing*, *13*(2), 93–110. <https://doi.org/10.1007/s10339-011-0430-z>

Hurst, C. D., Dunn, A. R., Dammer, E. B., Duong, D. M., Shapley, S. M., Seyfried, N. T., Kaczorowski, C. C., & Johnson, E. C. B. (2023). Genetic background influences the 5XFAD Alzheimer's disease mouse model brain proteome. *Frontiers in aging neuroscience*, *15*, 1239116. <https://doi.org/10.3389/fnagi.2023.1239116>

Masters, C. L., Bateman, R., Blennow, K., Rowe, C. C., Sperling, R. A., & Cummings, J. L. (2015). Alzheimer's disease. *Nature reviews. Disease primers*, *1*, 15056. <https://doi.org/10.1038/nrdp.2015.56>

Seibenhener, M. L., & Wooten, M. C. (2015). Use of the Open Field Maze to measure locomotor and anxiety-like behavior in mice. *Journal of visualized experiments : JoVE*, (96), e52434. <https://doi.org/10.3791/52434>

Vorhees, C. V., & Williams, M. T. (2006). Morris water maze: procedures for assessing spatial and related forms of learning and memory. *Nature protocols*, *1*(2), 848–858. <https://doi.org/10.1038/nprot.2006.116>
